# Machine learning and interactive GUI for concrete compressive strength prediction

**DOI:** 10.1038/s41598-024-66957-3

**Published:** 2024-07-19

**Authors:** Mohamed Kamel Elshaarawy, Mostafa M. Alsaadawi, Abdelrahman Kamal Hamed

**Affiliations:** 1 Civil Engineering Department, Faculty of Engineering, Horus University-Egypt, New Damietta, 34517 Egypt; 2 Structural Engineering Department, Faculty of Engineering, Mansoura University, Mansoura, 35516 Egypt

**Keywords:** Concrete, Compressive strength, Machine learning, SHAP analysis, k-fold cross-validation, Ensemble model, Prediction, Engineering, Civil engineering

## Abstract

Concrete compressive strength (CS) is a crucial performance parameter in concrete structure design. Reliable strength prediction reduces costs and time in design and prevents material waste from extensive mixture trials. Machine learning techniques solve structural engineering challenges such as CS prediction. This study used Machine Learning (ML) models to enhance the prediction of CS, analyzing 1030 experimental CS data ranging from 2.33 to 82.60 MPa from previous research databases. The ML models included both non-ensemble and ensemble types. The non-ensemble models were regression-based, evolutionary, neural network, and fuzzy-inference-system. Meanwhile, the ensemble models consisted of adaptive boosting, random forest, and gradient boosting. There were eight input parameters: cement, blast-furnace-slag, aggregates (coarse and fine), fly ash, water, superplasticizer, and curing days, with the CS as the output. Comprehensive performance evaluations include visual and quantitative methods and k-fold cross-validation to assess the study’s reliability and accuracy. A sensitivity analysis using Shapley-Additive-exPlanations (SHAP) was conducted to understand better how each input variable affects CS. The findings showed that the Categorical-Gradient-Boosting (CatBoost) model was the most accurate prediction during the testing stage. It had the highest determination-coefficient (R^2^) of 0.966 and the lowest Root-Mean-Square-Error (RMSE) of 3.06 MPa. The SHAP analysis showed that the age of the concrete was the most critical factor in the predictive accuracy. Finally, a Graphical User Interface (GUI) was offered for designers to predict concrete CS quickly and economically instead of costly computational or experimental tests.

## Introduction

Escalating emissions of Greenhouse Gases (GHG) have led to the decline of polar ice stores in the Arctic and Antarctic, presenting grave ecological challenges for the Earth^1^. The building industry currently stands as the predominant emitter of GHGs, contributing nearly half of the global emissions^2^. A key component of concrete, Portland cement (PC), plays a significant role in these emissions^3^. The fabrication of PC is responsible for approximately 7% of worldwide CO_2_ emissions, with half of this figure arising from the calcination process of calcium oxide (CaO)^4^. Current records show that PC production is at 4000 million tons annually, with projections suggesting an increase to 6000 million tons by 2060^5^. Such figures underscore the urgent need for effective substitutes that can satisfy the surging demand for concrete while reducing energy consumption and CO_2_ emissions^6^. One of the proposed scientific and viable solutions is to incorporate waste and recycled materials into concrete production, as it can offer a sustainable approach to meet the growing demand for concrete and simultaneously diminish environmental hazards and the degradation of our natural resources^7,8^.

Various alternative materials, sourced from by-products of industrial processes (such as ground granulated blast furnace slag (GGBS)^9^, granite powder^10^, and fly ash^11^), can serve as substitutes for PC. The incorporation of these alternative raw materials can enhance the qualities of concrete while simultaneously diminishing its ecological footprint. Research has demonstrated that replacing PC with materials like GGBS, granite powder, and fly ash in concrete can lead to a reduction of up to 80% in GHG emissions^12^. Typically, these industrial by-products are accumulated in large quantities, posing additional environmental concerns. Therefore, their utilization as a partial cement substitute represents an environmentally and industrially advantageous strategy^13^.

Concrete stands as the most utilized construction material on a global scale, surpassed only by water in terms of global consumption, owing to its valuable properties such as the availability of raw materials, ease of molding from liquid to solid states, and the capacity to bear loads post-hardening^14–17^. Its global utilization in structures is also due to its diverse advantageous characteristics, like compressive strength (CS), durability, stiffness, hardness, porosity, density, and resistance to fire and heat. Among these properties, compressive strength is considered paramount, as it significantly influences the safety and durability of concrete structures^18^. The variance in concrete’s compressive strength is attributed to its heterogeneous nature, which consists of a binder, aggregates, water, and admixtures^19^. These components and how they are combined influence the material’s ability to endure compressive stress, with factors such as the water-to-binder ratio, aggregate size, and binder type playing significant roles^20^. The complexity of this mixture makes it challenging to determine the compressive strength accurately.

Laboratory testing involves crushing samples of standard dimensions after a specific curing period, is the conventional method for evaluating compressive strength^21^, and is uniformly accepted worldwide. However, such tests are now deemed inefficient and not cost-effective, both expensive and time-intensive. To circumvent these limitations, some studies have introduced various empirical regression techniques to predict concrete’s compressive strength from its mix proportions^22–24^. Nonetheless, the intricate and highly non-linear relationships between the concrete’s constituents and its strength render the creation of exact regression models exceptionally difficult^25^.

In the past few years, significant progress has been made in the artificial intelligence (AI) sector, highlighted by the evolution of machine learning (ML) models, including Artificial Neural Network (ANN), Support Vector Machines (SVM), Gene Expression Programming (GEP), and deep learning (DL). This advancement has enabled the extensive use of these techniques across different areas, thereby equipping researchers with powerful tools to address and resolve intricate challenges more efficiently. Compared to conventional regression methods, ML is capable of developing predictive models through certain models that effectively manage regression issues and demonstrate remarkably high accuracy^26–30^.

The prediction of strength properties of fly ash-based concrete holds significant importance due to the environmental benefits and improved performance characteristics it offers. Fly ash, a by-product of coal combustion is a promising alternative. Its incorporation into concrete mitigates waste disposal issues and enhances the concrete’s mechanical properties. Predicting the compressive strength of fly ash-based concrete using ML models is crucial. It allows for optimizing mix designs, ensuring the concrete meets the required performance standards while minimizing environmental impact. The main factors affecting the compressive strength of concrete include the water-to-binder ratio, aggregate size, binder type, curing age, and the specific characteristics of the alternative materials used, such as fly ash. Addressing these factors by applying ML models can significantly improve the accuracy of strength predictions and optimize concrete mix designs, thereby enhancing performance and sustainability.

Analyzing extensive data from previous concrete mixtures using ML models allows for predicting concrete properties, optimizing material proportions, enhancing quality control, and projecting long-term durability. These models are adept at identifying patterns and connections within data, which leads to more accurate predictions and improved decision-making in the design of concrete mixes. Additionally, machine learning offers opportunities to increase the sustainability of concrete mix designs. Through ML models, optimal material combinations can be identified, which contribute to reducing waste, decreasing carbon emissions, and improving concrete structures’ overall lifespan and performance, considering factors like cost, availability, and environmental impact. Several researchers have investigated the application of machine learning in structural engineering, especially in predicting the compressive strength of concrete.

Chithra et al.^31^ effectively employed the ANN algorithm to forecast the compressive strength of high-performance concrete (HPC) that incorporates silica nanoparticles and copper slag. Similarly, a study by Nguyen et al.^32^ introduced four distinct machine-learning models to anticipate the compressive and tensile strength of HPC, highlighting the superior output accuracy of models based on gradient boosting regressor (GBR) and extreme gradient boosting (XGBoost). Furthermore, Kumar et al.^33^ gathered data from 120 sets to craft several predictive models for assessing the compressive strength of lightweight concrete (LWC), identifying the SVM model as the most efficient. Ashrafian^34^ successfully applied heuristic regression techniques to estimate fibrous concrete’s strength and ultrasonic pulse velocity. In another work, Zhang et al.^35^ utilized the random forest (RF) algorithm to foretell the uniaxial compressive strength of lightweight self-compacting concrete. They also conducted a significant analysis of eight input variables to determine their importance. Additionally, Aslam et al.^36^ built a GEP-based model to predict the compressive strength of high-strength concrete (HSC), utilizing a substantial dataset comprising 357 points. An overview of the different ML models adopted in past literature and the approaches used in the present investigation is delineated in Table 1.

**Table 1:** ML models adopted in the past literature.

Name of author	Concrete type	Dataset	ML Algorithm name	Ref
Li et al. (2023)	HPC	1030	Gradient boosting regression tree (GBRT)	^25^
Song et al. (2022)	HPC	471	Ensemble and non-ensemble ML approach	^37^
Huang et al. (2022)	HSC	225	Firefly Algorithm (FA) and Random Forest (RF)	^38^
Wang et al. (2022)	HPC	1030	XGBoost, RF, and SVR	^39^
Ahmad et al. (2021)	HPC	270	GEP, DT, and Bagging	^40^
Zhang and Zhao (2017)	UHPC	78	ANN	^41^
Yu et al. (2018)	HPC	1761	SVM	^42^
Al-Mughanam et al. (2020)	SCC	7	ANFIS	^43^
Bui et al. (2018)	HPC	1133	ANN	^44^
Al-Hashem et al. (2022)	Pozzolanic concrete	310	GEP and ANN	^45^
Golafshani et al. (2020)	OPC and HPC	2817	ANN and ANFIS	^46^
Aslam et al. (2020)	HSC	357	GEP	^36^
Das and Kashem (2024)	UHPC	626 and 317	XGBoost, LightGBM, hybrid XGBoost-LightGBM	^47^
Karim et al. (2024)	RHA-FA concrete	138	ANN, XGBoost, GBM	^48^
Kashem et al. (2024)	UHPC	810	AB, RF, GB	^49^
Kashem et al. (2024)	RHA-based concrete	1404	LGB, XGB, RF, hybrid XGB-LGB, hybrid XGB-RF	^50^
Paul et al. (2024)	RHA concrete	1212	CatBoost, GBM, CNN, GRU	^51^
Haque et al. (2024)	MPC composites	3108	CNN-LSTM, CNN-GRU, DTR-RFR, GBR-RFR	^52^
Kashem & Das (2024)	HSC	681	XGBR-BR, SVR-RFR, GBR-DTR	^53^
Islam et al. (2024)	HPC	2171	BiLSTM, CNN, GRU, LSTM	^54^
Shaban et al. (2024)	Recycled aggregate concrete (RAC)	105	MSSA-DE	^55^
Shaban et al. (2024)	Brick aggregate concrete (BAC)	132	ANFIS-PSO, ANFIS-GA, ANFIS-FFA	^56^

The novelty of this study lies in its comprehensive approach to utilizing machine learning models for predicting concrete CS, offering significant improvements in accuracy and practical applicability over traditional methods. The literature review confirms their successful application in similar engineering contexts, validating their effectiveness and appropriateness for the study’s objectives. The following are the authors’ specific contributions:

1. To introduce diverse ML models, including non-ensemble (regression-based, genetic programming, neural network, fuzzy-inference-system) and ensemble models (adaptive boosting, random forest, gradient boosting, categorical boosting), and assess their effectiveness in predicting concrete CS.
2. Using Bayesian Optimization (BO) for hyperparameter tuning significantly enhances predictive accuracy.
3. To conduct a detailed performance analysis across low, moderate, and high CS ranges.
4. To perform a sensitivity analysis using Shapley-Additive-exPlanations (SHAP) to determine the most influential parameters affecting CS prediction, offering insights for improving predictive accuracy.
5. To close the gap between complex computational predictions and practical real-world applications by creating a user-friendly GUI, highlighting the practical value of the study and its comprehensive approach to integrating theoretical model development with professional usability.

## Research objectives

**Figure 1:**
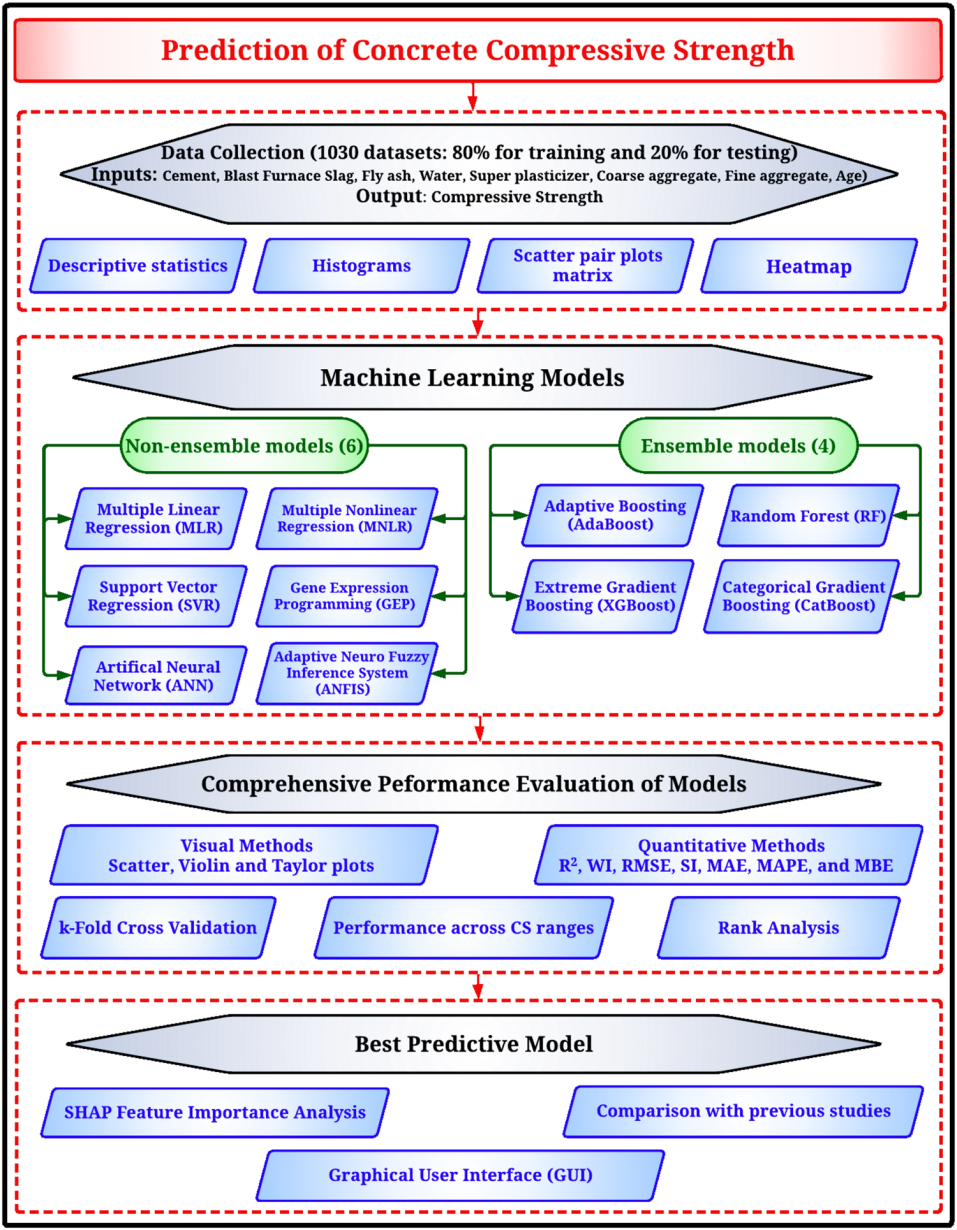
Methodology flowchart.

This research builds upon prior work by employing machine learning models to predict the CS of concrete across a broad spectrum of data, varying from 2.33 to 82.60 MPa. The main objective was to evaluate the efficacy of different ML models for predicting the CS of concrete. Figure 1 shows the flowchart of the methodological approach used in this study to predict the compressive strength of concrete. Initially, data from 1030 datasets are collected, including various components like cement and aggregates, and their properties are analyzed through histograms and heatmaps. Then, two types of predictive models are applied: non-ensemble models and ensemble models. The models’ performance is evaluated by comparing actual and predicted values, using metrics like R2 and RMSE, and through k-fold cross-validation. Sensitivity analysis is conducted, and the results are benchmarked against previous studies to identify the best predictive model. This approach aims to facilitate the researcher’s ability to gauge the effect of different variables on the prediction of CS in a more time-efficient and cost-effective manner compared to extensive experimental studies.

### Database collection

To develop a model for predicting outcomes and to analyze the data statistically, researchers can use data from laboratory experiments or gather information from previously published studies. For this research, a substantial dataset consisting of 1030 data points related to the CS of concrete was assembled by reviewing past scholarly articles: Song et al.^19^, Song et al.^37^, and Yeh^57^. The data analysis of the study focused on eight principal attributes, which were used as the input variables: cement (C), blast furnace slag (Slag), fly ash (FA), water (W), superplasticizer (SP), coarse aggregate (Cagg), fine aggregate (Fagg), and the number of days of curing (Age). These were all considered to predict the final compressive strength, the outcome variable. Table 2 provides a concise overview of the statistical description of the collected data, presenting a comprehensive summary of its characteristics. Each row refers to a distinct variable, while the columns contain specific statistical measures for these variables.

**Table 2:** Descriptive statistics for the collected database.

Variables	Symbol	Mean	Standard Deviation	Minimum	Median	Maximum	Count
C (kg m^−3^)	**X1**	281.17	104.5	102.0	272.9	540.0	1030
Slag (kg m^−3^)	**X2**	73.90	86.3	0.0	22.0	359.4	1030
FA (kg m^−3^)	**X3**	54.19	64.0	0.0	0.0	200.1	1030
W (kg m^−3^)	**X4**	181.57	21.4	121.8	185.0	247.0	1030
SP (kg m^−3^)	**X5**	6.20	6.0	0.0	6.4	32.2	1030
C_agg_ (kg m^−3^)	**X6**	972.92	77.8	801.0	968.0	1145.0	1030
F_agg_ (kg m^−3^)	**X7**	773.58	80.2	594.0	779.5	992.6	1030
Age (days)	**X8**	45.66	63.2	1.0	28.0	365.0	1030
CS (MPa)	**Y**	35.82	16.7	2.33	34.4	82.6	1030

**Figure 2:**
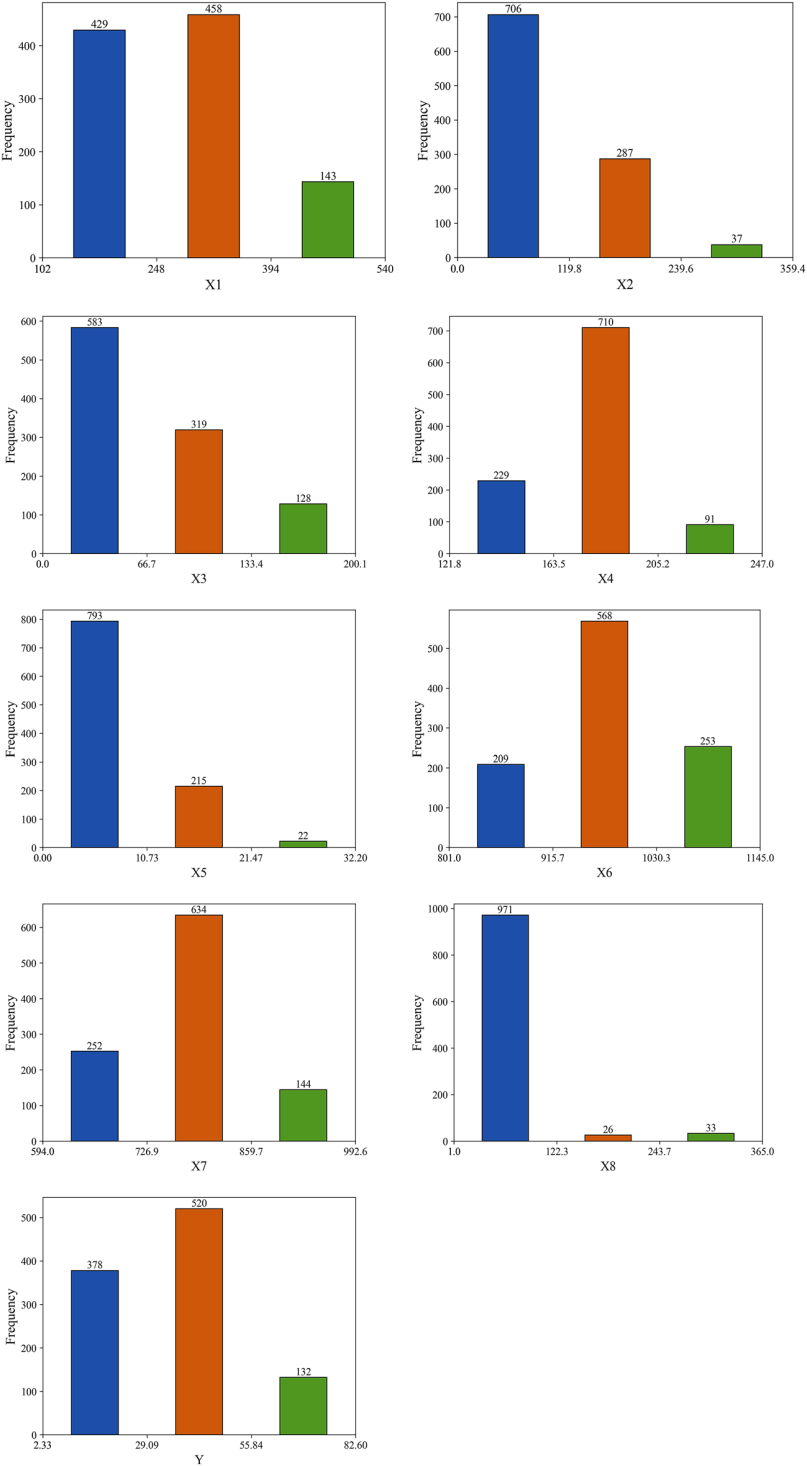
Histograms of input variables.

Furthermore, the frequency distribution of the dataset is visually represented in Fig. 2 through histogram plots. These plots are invaluable for understanding the distribution patterns of each variable, such as normality, skewness, and the presence of outliers, which align with the statistics presented in Table 2. The x-axis represents each variable, while the y-axis indicates the frequency of occurrences. This visualization enables a thorough assessment of these variables. The general observations include:

- Most variables (i.e., X2, X3, X5, and X8) show a robust positive skewness, indicating a higher concentration of lower values and fewer higher values.
- The X4, X6, X7, and Y variables display more balanced distributions with central tendencies.
- Outliers are more prominent in features with positive skewness, where higher values occur less frequently.

#### Correlation analysis

Examining the correlation between variables is crucial for comprehending the connections between dependent features and the target strength factor, as this analysis seeks to determine the most effective prediction model. This method’s most widely used measure is the Pearson correlation coefficient (*r*), which helps to understand these relationships^58,59^. It can be calculated as the ratio of the covariance (*cov*) of two variables (*x*, *y*) to the product of their standard deviations, as represented in Eq. (1).

1 $$r = \frac{{cov \left( {x,\;y} \right)}}{{\sigma_{x} \sigma_{y} }} = \frac{{\mathop \sum \nolimits_{i = 1}^{n} \left( {x_{i } - \overline{x}} \right)\left( {y_{i } - \overline{y}} \right)}}{{\sqrt {\mathop \sum \nolimits_{i = 1}^{n} \left( {x_{i } - \overline{x}} \right)^{2} } \sqrt {\mathop \sum \nolimits_{i = 1}^{n} \left( {y_{i } - \overline{y}} \right)^{2} } }}$$

where $\overline{x}$ and $\overline{y}$ are the mean of two variables *x* and *y*; *n* is the number of a dataset.

**Figure 3:**
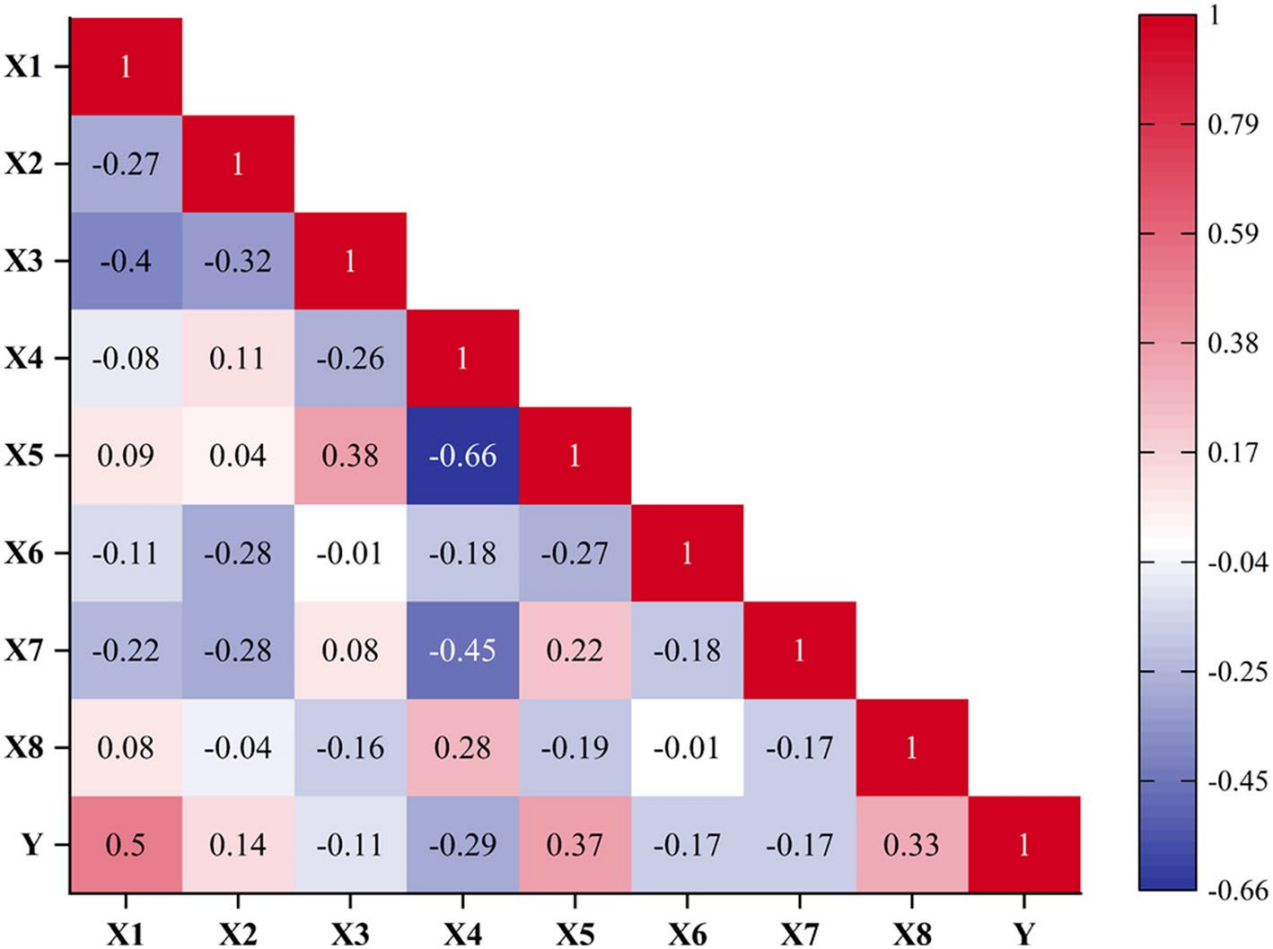
Pearson correlation of input and output variables.

Figure 3 presents a heatmap that demonstrates the influence of each variable on all other variables. Notably, the strongest positive correlations between X1, X5, X8, and Y are observed, with *r*-values of 0.50, 0.37, and 0.33, respectively. This indicates that the CS of concrete is significantly influenced by adding cement, followed by superplasticizer, and finally by the number of days of curing, as evidenced by their higher values. Conversely, the strongest negative correlation between X4 and X5 (− 0.66) suggests an inverse relationship between water and superplasticizers. Also, there is a substantial negative correlation between water and CS of concrete, with an *r*-value of − 0.29. The remaining variables show a weak correlation between concrete’s CS and each other, indicating that variables do not have linear solid relationships with each other. The absence of uncorrelated features implies that all eight input parameters are relevant and can be effectively employed in predicting the CS of concrete.

**Figure 4:**
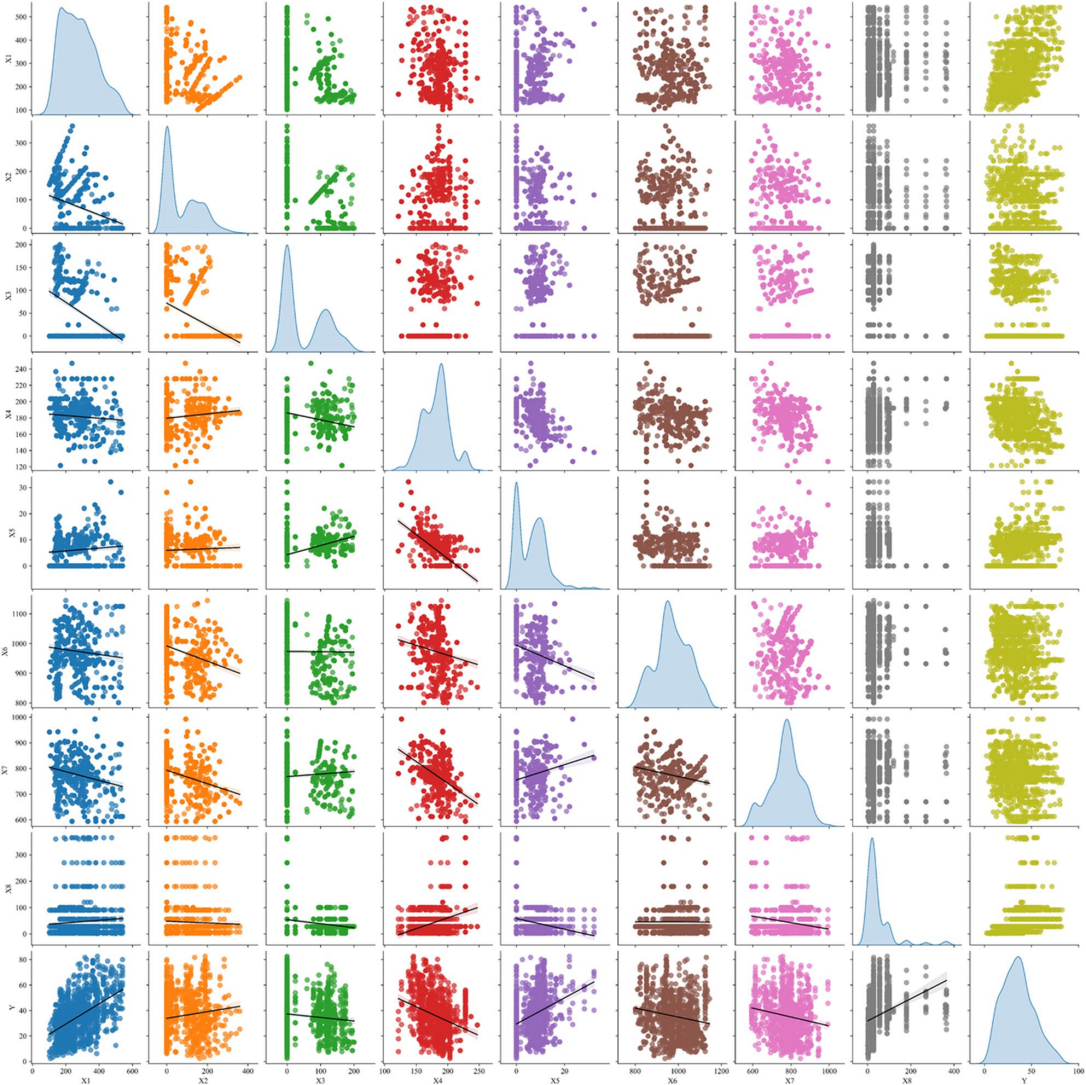
Scatter pair plots matrix with interaction variables.

Figure 4 presents a scatterplot matrix that provides a comprehensive visual analysis of the eight input variables and their relationship with the output variable (Y). The matrix includes histograms on the diagonal, illustrating the distribution of each variable individually, as previously discussed. The off-diagonal cells in the matrix contain scatter plots that show the pairwise relationships between variables. Each scatter plot provides a visual representation of the correlation between two variables. The input parameters X1 and X5 exhibit a clear positive linear relationship with the output variable Y in Positive Linear Relationships. These findings suggest a positive correlation between the increase in X1 and X5 and the increase in Y, indicating a direct relationship. In addition, the variables X3 and X5 exhibited a positive linear relationship, indicating that as the value of X3 increases, the value of X5 also tends to increase.

A strong negative linear relationship is evident between the input parameters X3 and X4, indicating that as X3 increases, X4 consistently decreases. Furthermore, the correlation between X4 and X5 is highly negative, suggesting that higher values of X4 correspond to lower values of X5. In contrast, the X2 input exhibits weak or unclear linear associations with the majority of other variables, suggesting a low level of correlation. Similar to X2, the input X6 does not exhibit distinct linear patterns with the majority of its variables. Regarding the input parameter X7, it is worth noting that while there is a minor correlation with X5, overall, X7 does not exhibit significant linear associations with most other variables. The input X8 exhibits clear clusters when plotted against other input parameters, indicating the existence of sub-groups within the data. Finally, a subtle non-linear pattern can be observed regarding the correlation between inputs X6 and X7. To gain a better understanding of the underlying relationship, additional investigation may be necessary.

#### Data normalization

Some machine learning models may not function optimally when there is a variation in the scale of input data. As indicated in Table 2, the cement range lies between 102 and 540 kg/m^3^, while the range for superplasticizers is between 0.0 and 32.2%, highlighting the disparate magnitudes of different input features. To address this, data normalization or rescaling is employed, which adjusts all input variables to a uniform scale. This process utilizes the max–min mapping function, as outlined in Eq. (2).

2 $$X_{n} = \frac{{X - X_{min} }}{{X_{max} - X_{min} }}$$

In this equation, *X*_*n*_ represents the normalized data, *X*_*min*_ and *X*_*max*_ denote the minimum and maximum values of each input variable, and *X* refers to the original dataset undergoing rescaling. The primary benefit of data rescaling lies in its ability to expedite computations and enhance the accuracy and stability of the machine learning-based prediction model.

### Non-ensemble models

In this research, six different non-ensemble models were utilized, namely Multiple Linear Regression (MLR), Multiple Nonlinear Regression (MNLR), Support Vector Regression (SVR), Gene Expression Programming (GEP), Artificial Neural Networks (ANN), and Adaptive Neuro-Fuzzy Inference System (ANFIS). These models were developed using the Python programming environment within the ANACONDA software, MATLAB, and SPSS programs. Concise explanations of each model are provided in the subsequent sub-sections.

#### MLR model

The MLR model is an extension of simple linear regression to predict a single output variable using multiple input variables^60–62^. This method assumes a linear relationship between the inputs and the output. It’s particularly valuable in situations where various factors influence the response variable, allowing for the assessment of the relative contribution of each predictor. The MLR model is straightforward, interpretable, and widely used in various fields for its ability to provide insights into relationships between variables. Multivariate Linear Regression is effective for predicting and understanding the underlying data structure.

#### MNLR model

Nonlinear models are straightforward, easy to understand, and effective for making predictions^63^. These models are versatile in terms of the range of average outcomes they can express. However, they might not be as adaptable as linear models when describing different data types. Nonetheless, if the nonlinear model is well-suited for a particular situation, it could be more efficient, use fewer parameters, and be simpler to understand. This clarity is often due to how parameters relate to significant, meaningful processes.

The process of using the MNLR model involves several steps: firstly, identifying the variable we want to predict; secondly, creating a nonlinear equation that represents how this variable is affected by other variables; thirdly, inputting initial guesses for the parameters of this equation, with the Levenberg–Marquardt method being the chosen technique for estimation; and finally, initiating the MNLR analysis to generate and review the results in the output log.

#### SVR model

The SVR model is an extension of Support Vector Machines (SVMs) used for regression problems^64,65^. SVR effectively finds the best-fit hyperplane in a high-dimensional space that can predict continuous values, maintaining a balance between the complexity of the model and the amount of error tolerated. It’s especially useful for datasets with many features and is known for its robustness against overfitting. This study uses the linear kernel to model relationships between input variables and the target variable linearly. The linear kernel in SVR essentially represents a straight line in the feature space. It assumes that the relationship between the input features and the target variable is linear, meaning that a change in the input features results in a proportional change in the predicted value.

#### GEP model

**Figure 5:**
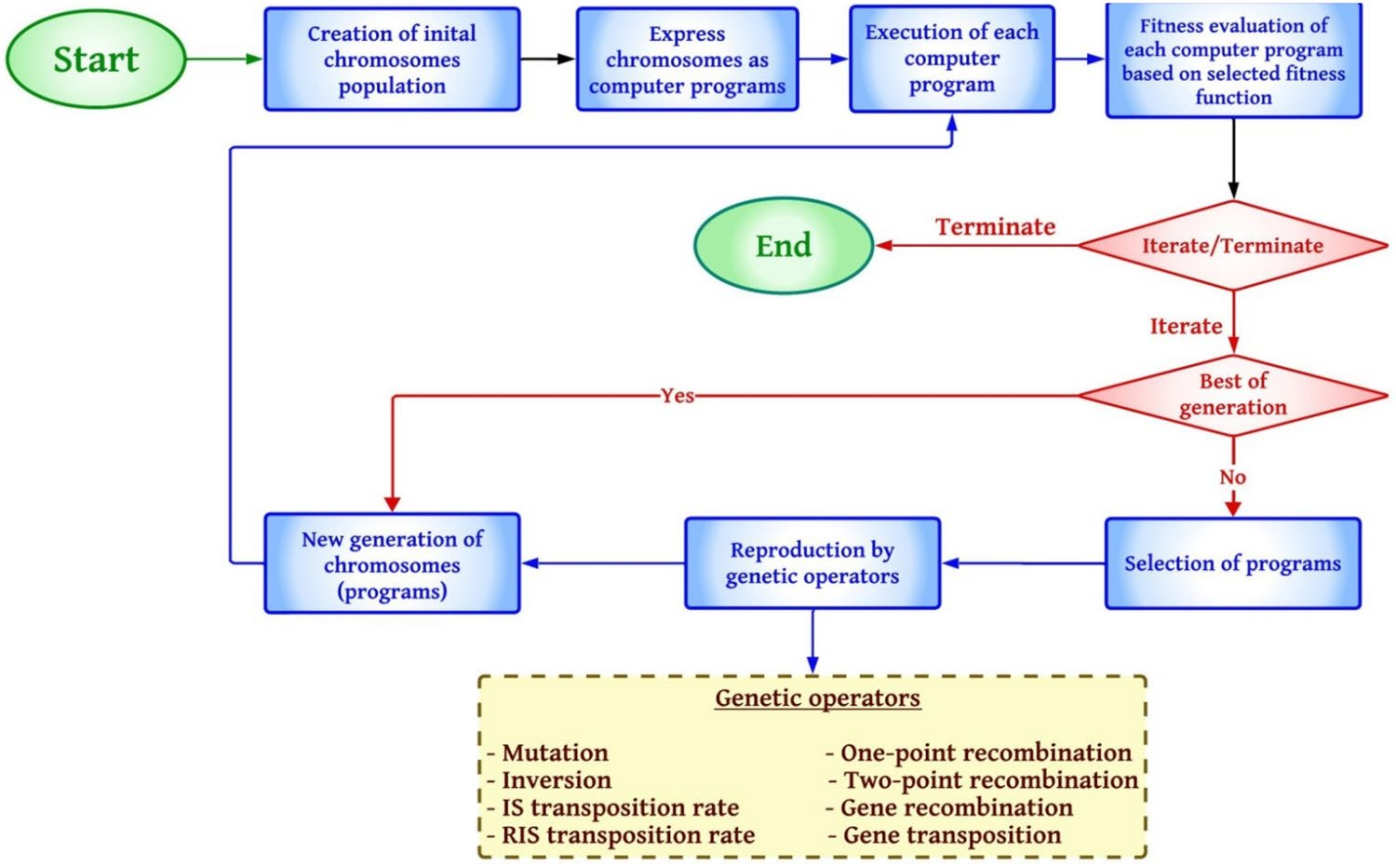
Flowchart of GEP model^67^.

The GEP model was developed to create computer programs and is similar to Genetic Algorithms (GAs) and Genetic Programming (GP)^61,66^. Figure 5 shows a flowchart of the GEP model. The GEP model follows a structured flow that begins with creating an initial chromosome population, representing potential solutions. These chromosomes are then expressed as computer programs. Following this, each program is executed, and its performance is evaluated based on a predefined fitness function.

If the termination condition is met, the process ends. Otherwise, it iterates to produce a new generation. This involves selecting the best-performing programs to continue to the next cycle and using genetic operators to create a new generation of chromosomes. These genetic operators include mutation, inversion, one-point recombination, two-point recombination, gene recombination, and insertion sequence (IS) transposition rate. The cycle repeats, continually evaluating the fitness of programs and generating new ones until the best possible solution is found or another termination condition is met, at which point the model concludes.

#### ANN model

**Figure 6:**
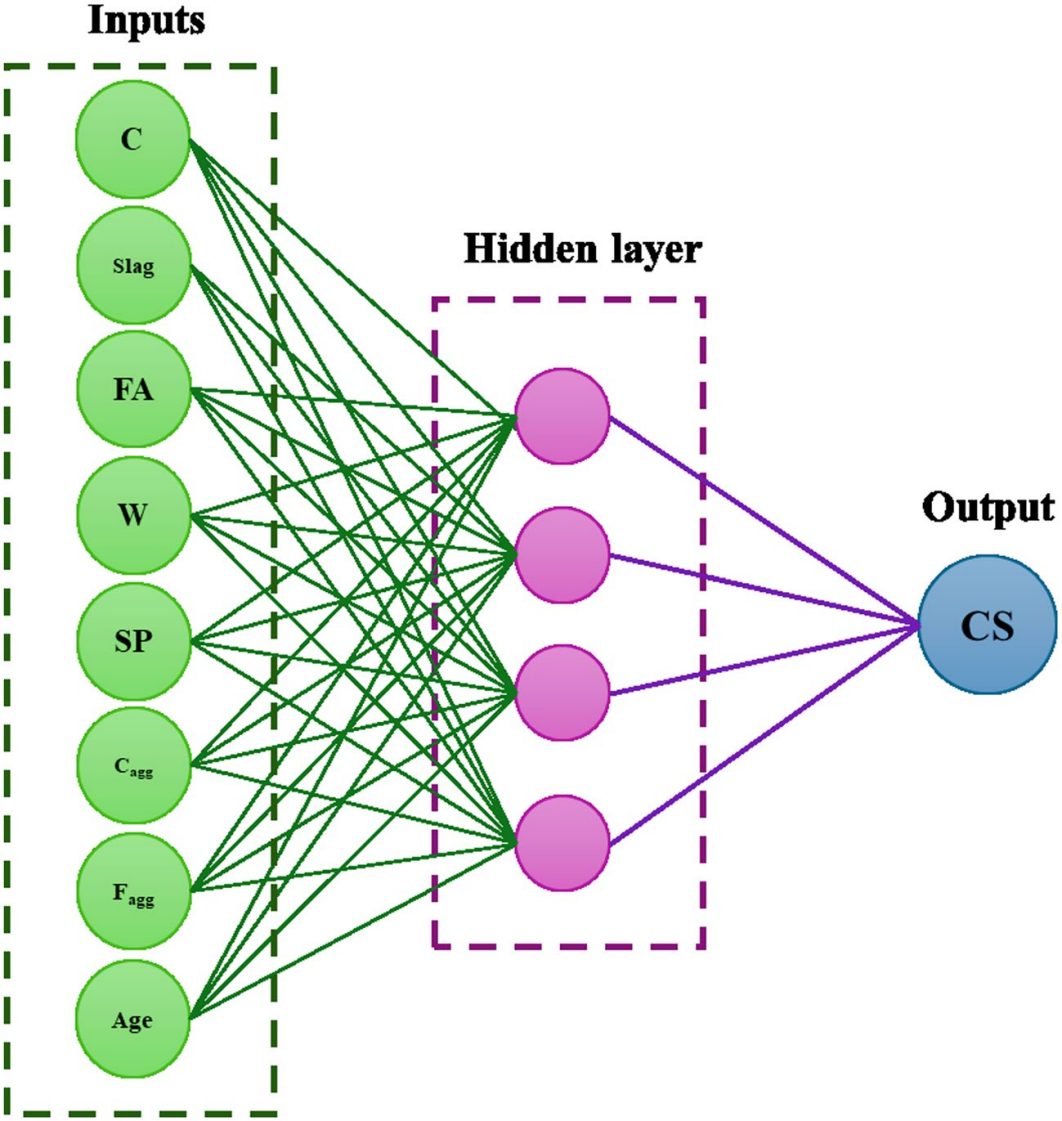
Inputs and output variables used for ANN model development.

ANNs are a cornerstone of machine learning, inspired by the structure and function of the human brain. They are particularly adept at identifying complex, non-linear relationships within large datasets. ANNs consist of interconnected nodes or neurons, which collectively learn to perform tasks like regression and classification by considering examples. Their flexibility and adaptability make them suitable for various applications, from image recognition to natural language processing^67–69^. A typical neural multilayer perceptron in an ANN consists of three layers: an input layer, one or more hidden layers, and an output layer, as illustrated in a three-layered architecture. In predicting new data sets, a model employs numerous neurons organized into a network to process information. These neurons are interconnected through weights and biases, crucial determinants of a machine learning model’s precision. Networks can be categorized into basic ANNs with a single hidden layer or deep neural networks with multiple layers. Utilizing additional hidden layers augments the ANN’s ability to identify the connections between inputs and outputs, thereby enhancing model accuracy. Figure 6 shows that the variable Y, represented by the CS of concrete, was set as the output from the ANN model, while the eight variables were assigned as the inputs to the ANN model.

#### ANFIS model

The ANFIS model, initially introduced by Jang^70^ and subsequently elaborated upon by Jang et al.^71^, constitutes a universal approximation methodology. In this capacity, it can approximate any real continuous function defined on a compact set with arbitrary precision. The ANFIS structure closely resembles an ANN, featuring five layers, each comprised of nodes, including rules. Notably, the Sugeno fuzzy model, as proposed by Takagi and Sugeno^72^, is frequently employed in ANFIS. A prototypical rule set for a first-order Sugeno fuzzy model, embodying two fuzzy If–Then rules, can be succinctly expressed as follows:

3 $${\text{Rule}}\;1:\;\;{\text{If}}\;x\;{\text{is}}\;A_{1} \;\;{\text{and}}\;\;y\;{\text{is}}\;B_{1} ,\;\;{\text{then}}\;f_{1} = p_{1} x + q_{1} y + r_{1}$$

4 $${\text{Rule}}\; 2:\;\;{\text{ If}}\; x\;{\text{ is}}\; A_{2} \;{\text{ and}}\; y\;{\text{ is}}\; B_{2} , \;{\text{then}}\; f_{2} = p_{2} x + q_{2 } y + r_{2}$$

**Figure 7:**
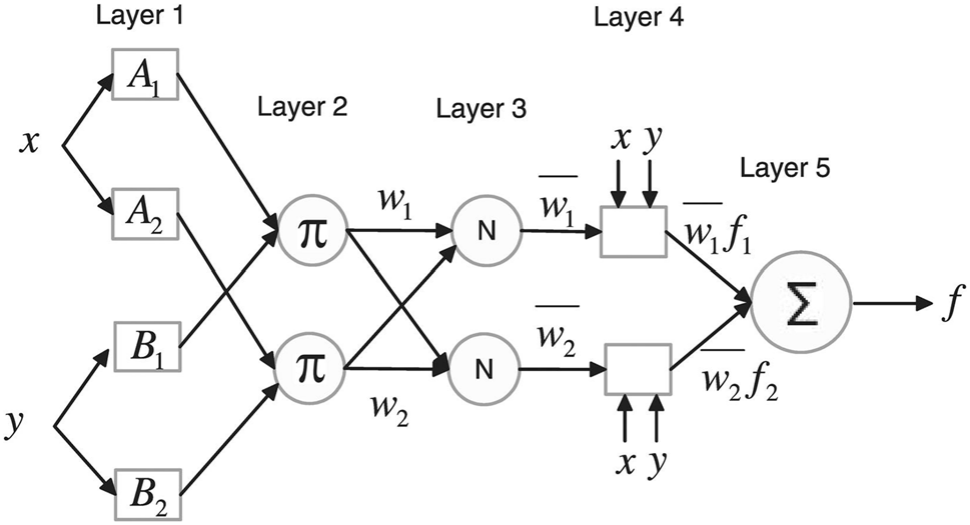
ANFIS architecture^74^.

Here, $(A_{1} ,\;A_{2} ,\;B_{1} ,\;B_{2} )$ represent fuzzy sets, and $(p_{ij} ,\;q_{ij} ,\;r_{ij} )$ denote parameters associated with the consequent part of each rule. This formulation describes the basic configuration of a Sugeno fuzzy model as it applies to an ANFIS. Figure 7 depicts the equivalent ANFIS framework. In this ANFIS setup, nodes within the same level perform similar functions. The structure is composed of five layers: the first is the input layer, followed by the rule layer, then the normalization layer, the consequent layer, and finally, the output layer. For an in-depth explanation of the ANFIS framework, one can refer to Chang and Chang^73^.

### Ensemble models

In this research, four different ensemble models were utilized, namely Adaptive Boosting (AdaBoost), Random Forest (RF), eXtreme Gradient Boosting (XGBoost), and Categorical Gradient Boosting (CatBoost). The development of these models was carried out using the Python programming environment within the ANACONDA program. Concise explanations of each model are provided in the subsequent sub-sections.

#### AdaBoost model

Boosting is a well-known algorithm in machine learning, first suggested by Schapire^75^. Subsequently, Freund^76^ developed AdaBoost. This method focuses on combining several basic classifiers created during training into a single strong classifier. Additionally, it enhances the training process to improve the formation of these basic classifiers. The AdaBoost model is an ensemble technique that combines multiple weak learners to form a strong learner. In regression, it sequentially fits a model to adjust the weights of instances based on the errors of the previous model, focusing more on difficult-to-predict instances. The AdaBoost model is often used to improve the accuracy of decision trees and is known for its simplicity and effectiveness in reducing bias and variance.

#### RF model

The RF model is an ensemble learning method that operates by constructing many decision trees during training and outputting the average prediction of the individual trees. Breiman^77^ first developed the RF model, which combines the ideas of randomly selecting features and grouping data samples together. The RF model is widely used for both classification and regression tasks. It is particularly well-known for its ability to handle large datasets with higher dimensionality and provides estimates of feature importance, which can be very insightful.

#### XGBoost model

The XGBoost model implements gradient-boosted decision trees designed for speed and performance. It is a highly flexible and versatile algorithm known for its efficiency in handling sparse data and its ability to perform well on a wide range of regression and classification problems. The XGBoost model has been used successfully in numerous machine learning competitions due to its scalability and ability to produce highly competitive predictive models^62^.

The choice to use XGBoost for this research is based on its useful characteristics. It uses regularization to avoid overfitting and uses second-order gradients for quicker convergence. It can handle missing data when finding splits and uses stochastic gradient descent to increase variety and reduce overfitting. Reduction is also employed to minimize overfitting. Furthermore, the XGBoost is designed with system-level enhancements such as parallel processing and cache optimization, which make it both fast and capable of handling large datasets.

#### CatBoost model

Categorical Gradient Boosting (CatBoost) represents a recent advancement in gradient-boosting algorithms designed to handle categorical features while minimizing information loss^78^. CatBoost distinguishes itself through two key characteristics: the utilization of ordered boosting to mitigate target leakage and its effectiveness, particularly on small datasets. Within the CatBoost model, the computation of the sample average for $x_{{\sigma_{i,k} }}$ involves considering the target values of preceding samples in a random permutation: $\sigma$ = ($\sigma_{1}$, $\sigma_{2}$, …, $\sigma_{N}$) of the dataset, thereby guarding against overfitting. This process is illustrated by Eq. (5).

5 $$x_{{\sigma_{i,k} }} = \mathop \sum \limits_{j = 1}^{i - 1} \left[ {x_{{\sigma_{i,k} }} = x_{{\sigma_{j,k} }} } \right]y_{{\sigma_{j} }} + \overline{a} P/\mathop \sum \limits_{j = 1}^{i - 1} \left[ {x_{{\sigma_{i,k} }} = x_{{\sigma_{j,k} }} } \right]y_{{\sigma_{j} }} + \overline{a}$$

where $x_{{\sigma_{i,k} }} = x_{{\sigma_{j,k} }}$ denotes a condition being met, indicated by a value of 1. *P* represents a predetermined value, and $\overline{a}$ is the coefficient to determine the significance of *P*.

### Hyperparameters tuning

**Figure 8:**
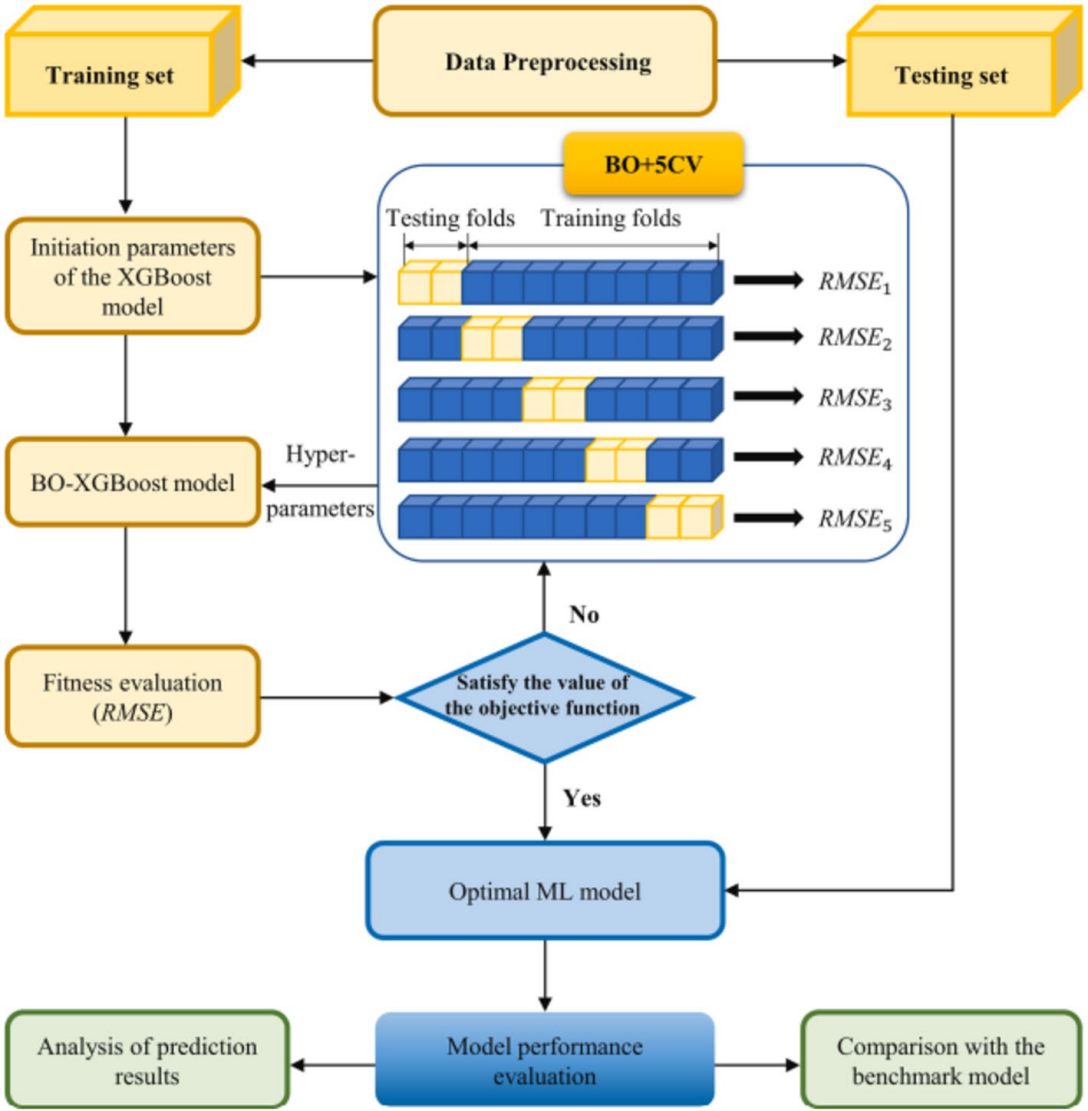
Framework example of XGBoost with Bayesian optimization^83^.

To optimize the hyperparameter settings in the current study, BO is employed. This technique contrasts with traditional methods like Grid-Search by initially modeling the prior distribution of the objective function and iteratively refining the search within the hyperparameter space for the optimal configuration. Initially, each model’s range and prior distribution of hyperparameters are specified. BO then identifies the configuration that maximizes performance within this predefined hyperparameter space. This approach enhances the efficiency of hyperparameter tuning, minimizing redundant experiments and accelerating the identification of the most effective hyperparameter combinations^79–82^. Figure 8 illustrates a framework example of BO-XGBoost.

### Comprehensive performance evaluation of models

The dataset was methodically partitioned into two distinct sets: training and testing. The training set is used to fit the model, and the testing set is used to evaluate the model’s predictive performance. The split ratio was carefully chosen to balance the need for sufficient training data against the necessity of a robust evaluation. Hence, the collected dataset was divided into 80% for training and 20% for testing. This ensures that the model is trained on a representative sample of the data while still subject to rigorous testing on data it has not previously encountered. However, two commonly used approaches involve quantitative and visual methods to evaluate and compare the adopted ML models^84^.

#### Visual methods

Visual methods include scatter plots, violin boxplots, and Taylor diagrams. These methods offer a quick and informative way to compare models, providing insights into accurate predictions for various statistical measures like maximum, minimum, median, and quartiles. They may not capture information about the performance and ranking of models. Scatter plots are used to visualize the relationship between two variables. Violin plots provide a full distribution of the data. This is crucial when comparing models because it shows not only the central tendency (i.e., mean or median) but also the spread and density of model performance metrics. Taylor diagrams are a specialized graphical representation that quantifies the similarity between actual and predicted values. These diagrams plot the correlation, the standard deviation, and the root mean square error of predictions on a single chart. This provides a comprehensive view of a model’s accuracy, variability, and overall performance compared to the actual observations.

#### Quantitative methods

Quantitative methods including seven performance indices: Determination Coefficient (R^2^), Willmott Index (WI), Root Mean Square Error (RMSE), Scatter Index (SI), Mean Absolute Error (MAE), Mean Absolute Percentage Error (MAPE) and Mean Bias Error (MBE). The ideal values for these indices are as follows: R^2^ and WI should ideally be 1, indicating perfect prediction accuracy, while RMSE, SI, MAE, MAPE, and MBE should ideally be 0, indicating no error in the predictions. In summary, a predictive model is ideal if its performance indicators are close to or strictly at these values. The equations for calculating these indices are presented in Eqs. (6–12) as follows^85^:

6 $$R^{2} = 1 - \frac{{\mathop \sum \nolimits_{i = 1}^{n} \left( {x_{i} - y_{i} } \right)^{2} }}{{\mathop \sum \nolimits_{i = 1}^{n} \left( {x_{i} - \overline{x}} \right)^{2} }}$$

7 $${\text{WI}} = 1 - \frac{{\mathop \sum \nolimits_{i = 1}^{n} \left( {x_{i} - y_{i} } \right)^{2} }}{{\mathop \sum \nolimits_{i = 1}^{n} \left( {\left| {x_{i} - \overline{x}} \right| + \left| {y_{i} - \overline{x}} \right|} \right)^{2} }}$$

8 $${\text{RMSE}} = \sqrt {\frac{{\mathop \sum \nolimits_{i = 1}^{ n} \left( {x_{i} - y_{i} } \right)^{2} }}{n} }$$

9 $${\text{SI}} = \frac{RMSE}{{\overline{{x_{i} }} }}$$

10 $${\text{MAE}} = \frac{1}{n} \mathop \sum \limits_{i = 1}^{ n} \left| {x_{i} - y_{i} } \right|$$

11 $${\text{MAPE}} = \frac{1}{n} \mathop \sum \limits_{i = 1}^{ n} \left| {\frac{{x_{i} - y_{i} }}{{x_{i} }}} \right|$$

12 $${\text{MBE}} = \frac{1}{n} \mathop \sum \limits_{i = 1}^{n} (x_{i} - y_{i} )$$

where $x_{i}$ is the actual CS values; $\overline{{x_{i} }}$ is the mean of the actual CS dataset; $y_{i}$ is the predicted CS value.

#### k-fold cross-validation

**Figure 9:**
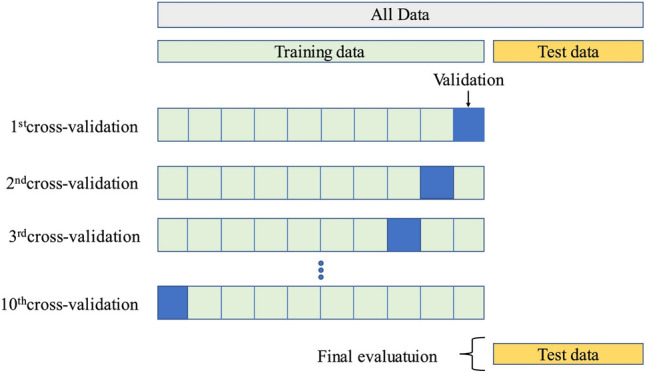
Schematic representation of k-fold cross-validation^86^.

K-fold cross-validation (Fig. 9) is a widely used method to check the performance of ML models. It involves dividing the dataset into several parts, typically ten, known as “folds.” In this tenfold system, the dataset splits into ten subsets. For each test, nine groups are used to train the model, and one group is kept for testing. This approach is suitable for understanding variability within the data and doesn’t take too much time to compute^62^. Each of the ten subsets becomes the test set, with the others being used for training. A reliable measure of model accuracy is obtained by averaging results from all ten tests. This way of testing helps ensure effective training of the adopted ML models and reduces the chance of missing out on essential data in the dataset.

#### SHAP feature importance analysis

**Figure 10:**
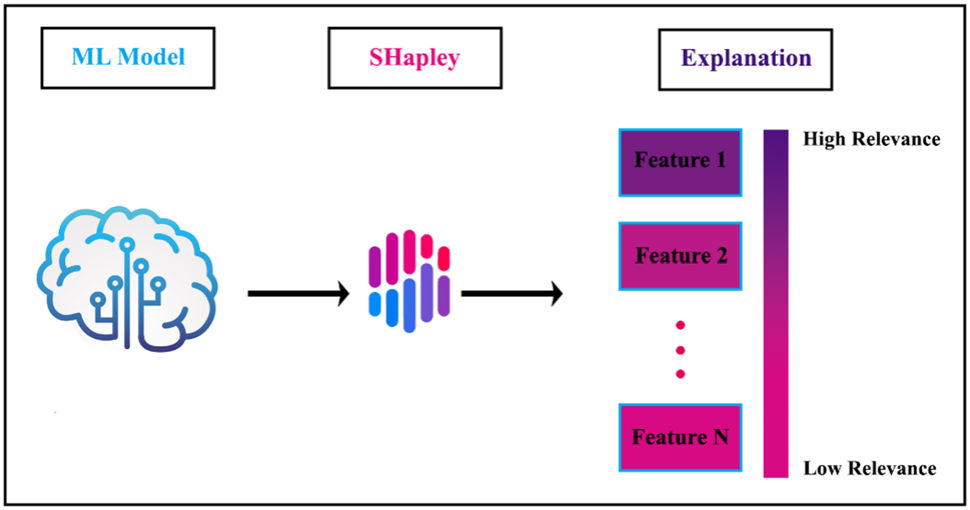
SHAP values method workflow.

To analyze the sensitivity and interpret ML models on both a wide-scale and a more detailed level, researchers use the SHAP approach, which draws on principles from cooperative game theory^47^. The SHAP method was employed to gauge the comparative impact of input variables on the prediction process. As an advanced method within the realm of explainable artificial intelligence, SHAP helps clarify the complex interactions between the input variables and the model predictions, as shown in Fig. 10. It offers critical insights by identifying which features are most influential on predictions and how they modify the predicted results^87,88^. Equation (13) shows the Shapley value $\phi_{i}$ for feature *i* is determined by calculating the average marginal contribution of that feature across all possible permutations of features. In this equation, *N* represents the set of all features, *S* represents a subset of features that excludes feature *i*, ∣*S*∣ denotes the cardinality of set (*S*), *v*(*S*) represents the model’s prediction when only features in set (*S*) are considered, and *v*(*S* ∪ {*i*}) represents the model’s prediction when feature *i* is added to set *S*^89^*.*

13 $$\phi_{i} = \mathop \sum \limits_{{S{ \subsetneq }N\left\{ i \right\}}} \frac{{\left| s \right|!\left( {\left| N \right| - \left| S \right| - 1} \right)!}}{\left| N \right|!}\left[ {v\left( {S \cup \left\{ i \right\}} \right) - v\left( S \right)} \right]$$

## Results and discussion

### Hyperparametric configuration of ML models

The optimal form of the equations developed by the MLR and MNLR models was the result of extensive testing. Considering the developed MLR model, the derived linear equation (Eq. 14) from the MLR model can be written as follows:

14 $${\mathbf{Y}} = - 23.331 + 0.120 {\mathbf{X1}} + 0.104 {\mathbf{X2}} + 0.088 {\mathbf{X3}} - 0.150 {\mathbf{X4}} + 0.292 {\mathbf{X5}} + 0.018 {\mathbf{X6}} + 0.020 {\mathbf{X7}} + 0.114 {\mathbf{X8}}$$

Regarding the developed MNLR model, the derived nonlinear equation (Eq. 15) from the MNLR model can be written as follows:

15 $${\mathbf{Y}} = \left[ {\left( {{ }{\mathbf{X1}}^{0.097} - 0.89 {\mathbf{X2}}} \right){*}\left( {{\mathbf{X2}}*{\mathbf{X5}}*{\mathbf{X7}}} \right)^{{ - 1.17 \times 10^{ - 4} }} { }} \right]{* }\left[ {\left( {{ }{\mathbf{X1}}{*}{\mathbf{X3}}{*}{\mathbf{X8}}^{{4.4 \times 10^{ - 25} }} } \right){*}\left( {\frac{{{\mathbf{X8}}}}{{{\mathbf{X4}}}}} \right)^{0.94} { }} \right]{*}\left[ {\frac{{\left( {{ }{\mathbf{X6}}^{5.9} {* }{\mathbf{X3}}^{ - 0.88} } \right){*}\left( {{\mathbf{X1}} + {\mathbf{X3}} - 34.37} \right)}}{{\left( {14.3{ }{\mathbf{X4}}} \right)^{0.94} + {\mathbf{X2}}}}{ }} \right]$$

On the other hand, a trial-and-error approach was used to determine the hyper-parameters, architectures, and functions of the remaining ML models during the training stage. The model with the highest average prediction accuracy across the entire training set was chosen based on these results. The grid search hyper-parameter tuning for the developed ML models is shown in Table 3.

**Table 3:** Optimized primary hyperparameters for the adopted ML models.

Model	Optimal Hyperparameter Value
SVR	Box-Constraint: 94.114, Kernal type: linear, Kernel-Scale: 997.22
GEP	Number of chromosomes: 100, Head size: 8, Number of Genes: 5, Linking Functions: Multiplication, Literals: 24, Gene size: 58, Function Set: (+), (-), (*), (/); Exponential (Exp); Square, cube, quartic, quintic roots (Sqrt, 3Rt, 4Rt, 5Rt); (Ln); Addition with 3 and 4 inputs (add3, add4); Multiplication with 3 and 4 inputs (Mul3, Mul4); Input X to the power of 2, 3, 4, 5
ANN	No. of hidden layers: 3, No. of hidden neurons per layer: 20, Hidden layer activation function: Sigmoid, Output layer activation function: Sigmoid
ANFIS	FIS type: Sugeno, Type of membership function: gaussmf, Number of memberships: 19
AdaBoost	Number of estimators: 715, Learning rate: 0.42, Loss: Linear
RF	Number of estimators: 313, Maximum depth: 23, Min samples split: 2.0, Min samples leaf: 1.0
XGBoost	Number of estimators: 493, Learning rate: 0.067, Maximum depth: 4.0, Colsample bytree: 0.724, Sub sample: 0.742
CatBoost	Learning rate: 0.124, Maximum depth: 4.0, L2 leaf reg: 1.0

### Evaluation of the adopted models

#### Visual methods

**Figure 11:**
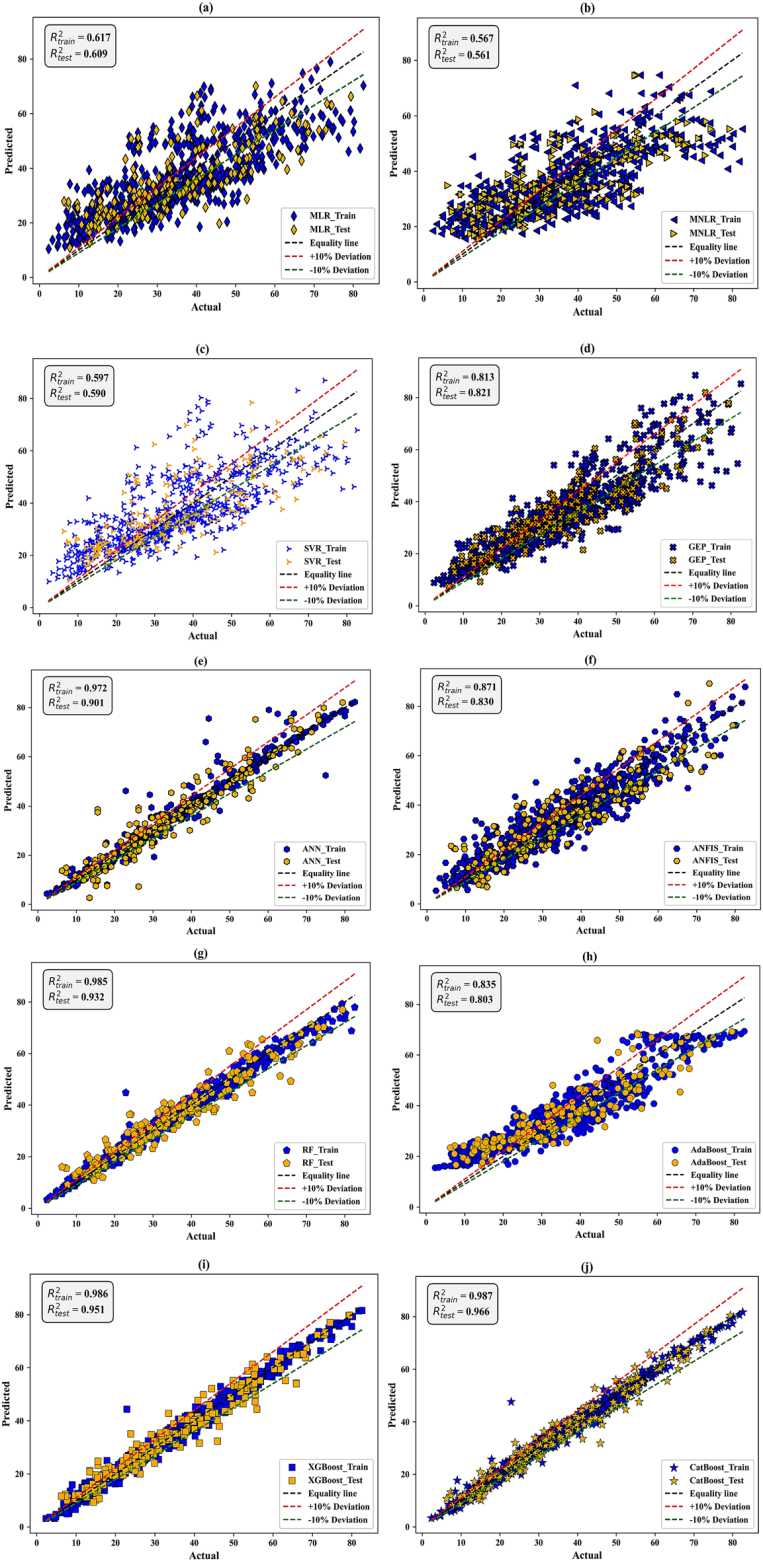
Scatter plots between predicted and actual CS values in the training and testing stages based on (**a**) MLR, (**b**) MNLR, (**c**) SVR, (**d**) GEP, (**e**) ANN, and (**f**) ANFIS, (**g**) RF, (**h**) AdaBoost, (**i**) XGBoost, and (**j**) CatBoost models.

The predictive accuracy of the adopted models for concrete’s CS was evaluated using visual methods: scatter plots, violin boxplots, and Taylor diagrams during the testing stage. Figure 11 shows ten scatter plots indicating the predicted versus actual values for training and testing stages across the adopted models. The distribution of most values along the black line, representing a perfect prediction with 0% error, clearly demonstrates that the model exhibits a high degree of precision in its predictions. Furthermore, the figures were elaborated with green and red boundary lines that indicate a ± 10% margin of error. This range includes the majority of projections, demonstrating the model’s resilience and reliability.

When evaluating the performance of individual models such as MLR, MNLR, and SVR, it is observed that the data points are widely dispersed and deviate significantly from the equality line. Many points fall beyond the ± 10% deviation lines in the training and testing stages. Given that the R^2^-values for these models were below 0.62, it can be concluded that these models exhibited poor predictive performance for the CS-value of concrete. The GEP model shows a distribution of data points that are scattered, with some clustering aligning along the line of equality. Approximately half the points are positioned above or below the ± 10% margin. The R^2^ values of the GEP model were 0.81 and 0.82 for the training and testing stages, respectively. This suggests that the model exhibited a moderate level of performance with a noteworthy degree of variability. Similar to the GEP model, the ANFIS model shows an acceptable level of performance, slightly surpassing the GEP model. The R^2^ values of the ANFIS model are 0.87 and 0.83 in the training and testing stages, respectively. Lastly, the ANN model outperformed all the non-ensemble models, achieving R^2^ values of 0.97 and 0.90 in the training and testing stages, respectively. This is evident from the fact that most data points are within a range of ± 10%.

Considering the performance of ensemble models, the AdaBoost model had the lowest predictive performance with R^2^ of 0.83 and 0.80 in the training and testing stages, respectively. The GEP model is a close equivalent of the AdaBoost model’s performance. In contrast, the CatBoost model had the highest accuracy, with R^2^ values of 0.987 and 0.966 in the training and testing stages, respectively. Following the CatBoost model, the XGBoost and RF present high predictive performance, with tight clustering data points and high R^2^ values (i.e., R^2^ > 0.98 in training and R^2^ > 0.93 in testing). In summary, ensemble models demonstrate superiority over non-ensemble models regarding the visual distribution of predicted versus actual values. Furthermore, the CatBoost is the best-performing model among all those that have been implemented.

**Figure 12:**
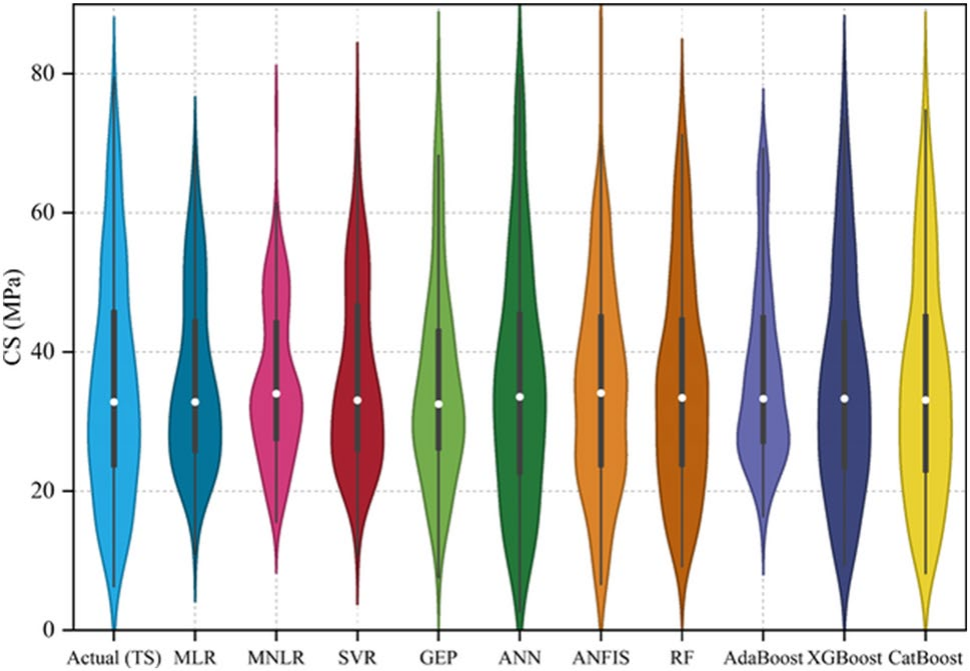
Violin boxplot for the performance of the adopted ML models in the testing stage.

**Figure 13:**
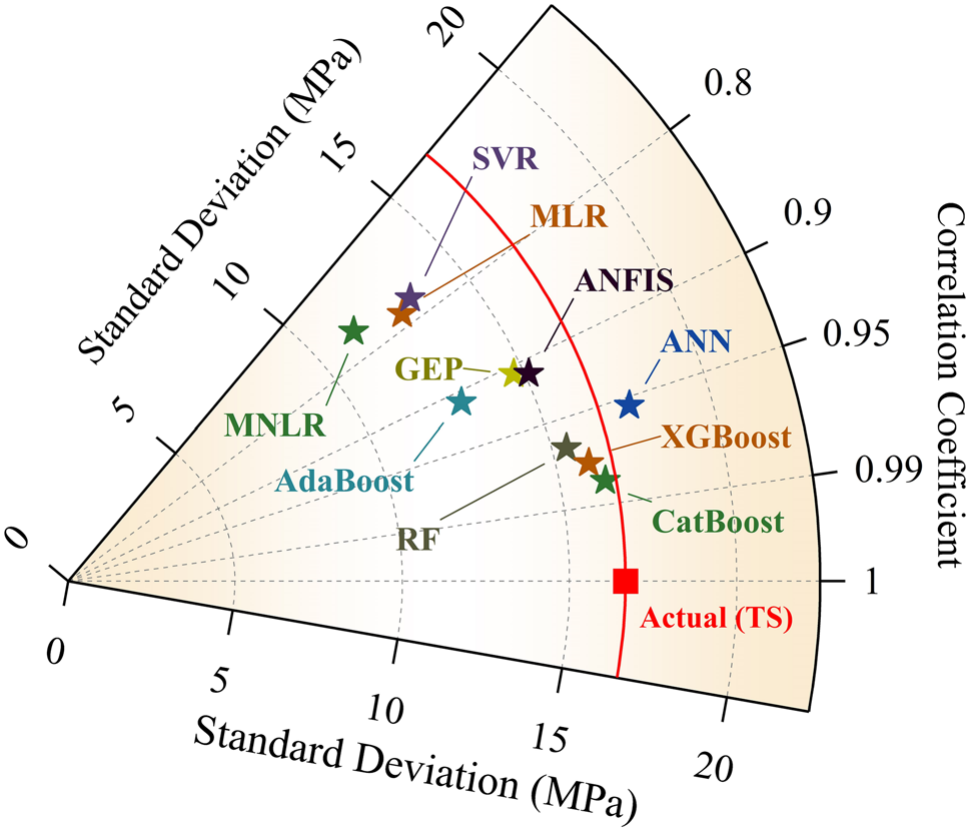
Taylor diagram for the performance of the adopted ML models in the testing stage.

Figure 12 shows the violin boxplots of the actual and predicted values during the testing stage (TS). This figure highlights the dataset’s minimum, maximum, median, ¼th, and ¾th percentile quartiles and overall distribution (actual and predicted values). It is evident that the shape model that most closely matches the actual dataset is the CatBoost model. Additionally, Fig. 13 shows a comparative analysis of the adopted models done using the Taylor diagram in the testing stage. Taylor’s diagram visually represents the degree of correspondence between a pattern and observed data. This diagram shows the correlation coefficient along one axis, the standard deviation as the distance from the center, and the centered Root-Mean-Square-Difference (RMSD) from the reference point (i.e., actual). Closer proximity of points to the reference indicates superior model performance. The models positioned along the arc with higher correlation coefficients (approaching 1.0) and smaller distances from the reference (lower RMSD) exhibit better performance. The CatBoost and XGBoost models were found to be the closest to the actual point. Meanwhile, the MNLR, MLR, and SVR models were found to be the furthest from the actual point.

#### Quantitative methods

Tables 4 and 5 show the seven performance indices calculated for the studied ML models for the training and testing stages, respectively. The tables show that the ensemble models were significantly better at making predictions than the individual non-ensemble models. Considering the bold values highlighted as the best predictive models in both stages, the best overall model would be the CatBoost model.

**Table 4:** Estimated performance indices for the studied ML models in the training stage.

Type	Model	R^2^	WI	RMSE (MPa)	SI	MAE (MPa)	MAPE	MBE (MPa)
Non-ensemble	MLR	0.617	0.870	10.34	0.151	8.23	0.313	0.132
	MNLR	0.567	0.836	11.00	0.186	8.54	0.357	− 0.319
	SVR	0.597	0.867	10.61	0.138	8.11	0.310	− 0.56
	GEP	0.913	0.946	7.21	0.090	5.66	0.201	− 0.072
	ANN*	**0.972**	**0.993**	**2.77**	**0.036**	**1.17**	**0.042**	− **0.194**
	ANFIS	0.871	0.965	6.00	0.073	4.61	0.172	− 0.001
Ensemble	RF	0.985	0.996	2.05	0.027	1.32	0.046	0.077
	AdaBoost	0.835	0.946	6.80	0.126	5.63	0.258	− 1.100
	XGBoost	0.986	0.996	2.00	0.026	1.38	0.050	− 0.012
	CatBoost*	**0.987**	**0.997**	**1.93**	**0.025**	**1.33**	**0.048**	− **0.001**

*The bold values indicated the best predictive models.

**Table 5:** Estimated performance indices for the studied ML models in the testing stage.

Type	Model	R^2^	WI	RMSE (MPa)	SI	MAE (MPa)	MAPE	MBE (MPa)
Non-ensemble	MLR	0.609	0.865	10.40	0.175	8.13	0.312	− 0.530
	MNLR	0.561	0.831	11.02	0.186	8.76	0.342	− 0.480
	SVR	0.590	0.862	10.65	0.157	8.16	0.307	− 1.026
	GEP	0.821	0.948	7.03	0.094	5.60	0.198	− 0.316
	ANN*	**0.901**	**0.976**	**5.24**	**0.066**	**3.51**	**0.135**	− **0.255**
	ANFIS	0.830	0.951	6.85	0.083	5.45	0.208	0.613
Ensemble	RF	0.932	0.981	4.35	0.067	3.12	0.117	− 0.017
	AdaBoost	0.803	0.935	7.39	0.140	5.96	0.255	− 1.485
	XGBoost	0.951	0.987	3.69	0.052	2.63	0.098	− 0.073
	CatBoost*	**0.966**	**0.991**	**3.06**	**0.042**	**2.27**	**0.083**	**0.129**

*The bold values indicated the best predictive models.

Considering evaluating non-ensemble models, the ANN consistently outperforms other non-ensemble models in both the training and testing stages. It obtains the highest R^2^ and WI values and has the lowest error indices, including RMSE, MAE, and MAPE. Meanwhile, the GEP and ANFIS exhibit a satisfactory level of performance, characterized by relatively high R^2^ and WI values, as well as moderate error indices. Conversely, based on the lowest R^2^ and WI indices and higher errors, the MLR, MNLR, and SVR models were the worst predictive models in both stages.

Among the ensemble models, the CatBoost model demonstrated superior performance during the training stage, achieving perfect scores in R2 and WI. Furthermore, it maintained excellent performance during the testing stage. Also, the XGBoost and RF models exhibit great predictive performance, as indicated by their high R^2^ and WI values and low error metrics. Although the AdaBoost model demonstrates satisfactory performance, it ranks slightly behind other ensemble models, especially during the testing stage. In general, ensemble models tend to exhibit superior performance compared to non-ensemble models. The CatBoost model is the best-performing model overall, followed closely by the XGBoost and RF models. Among individual models, the ANN model exhibit superior performance, making them a strong candidate in the absence of the ensemble models.

#### k-fold cross-validation

**Figure 14:**
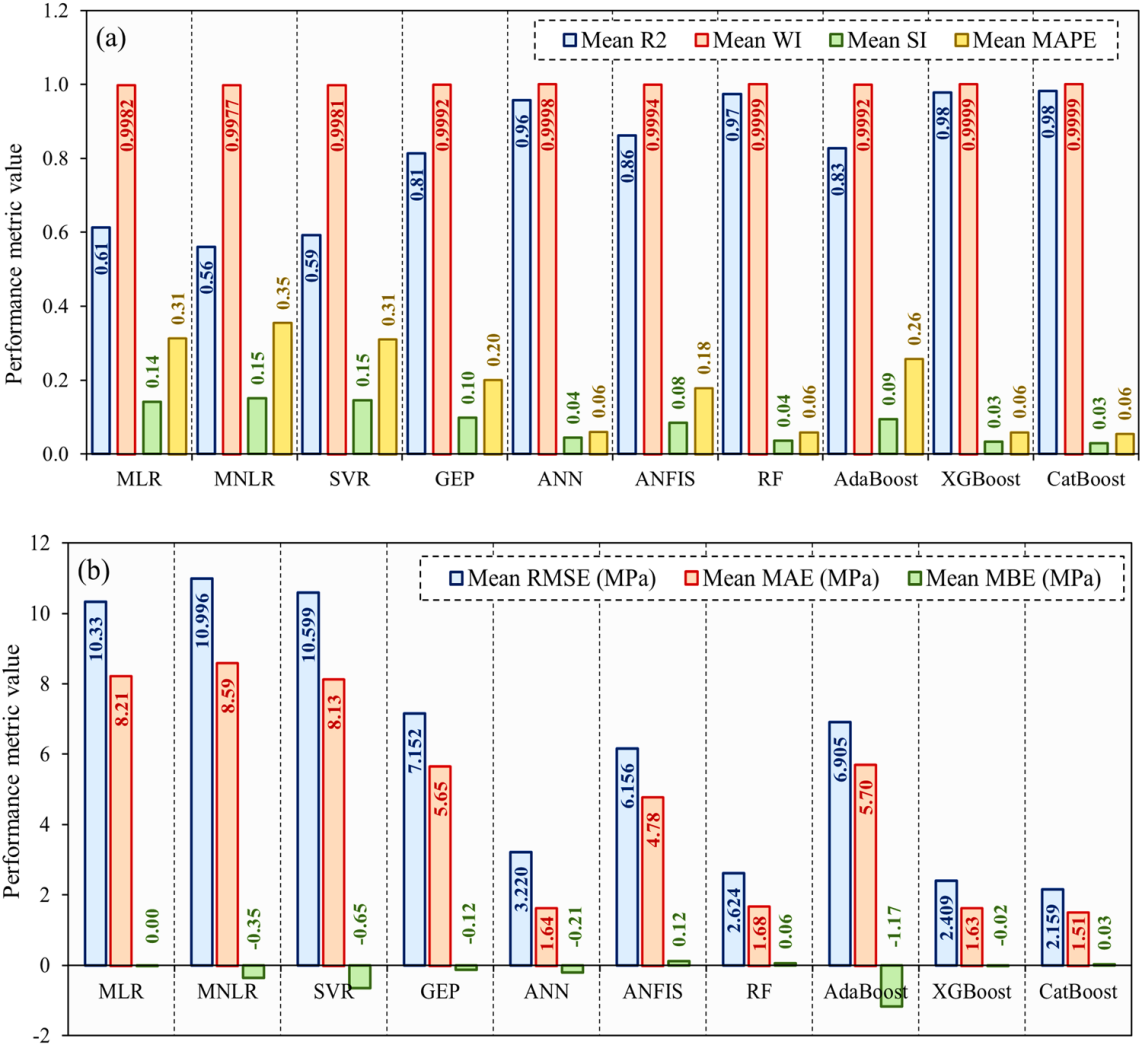
Comparison of average values of performance indices based on BO + 10 folds cross-validation process.

Utilizing k-fold cross-validation reduces the risk of models fitting too specifically to one part of the dataset, thereby providing a more accurate evaluation of their effectiveness. This method is typically used to refine models, aiming for a more precise measurement of performance and lowering the risk of overfitting to a singular train-test split. The outcomes from this method support the trustworthiness and accuracy of the evaluated models. Figure 14 compares the adopted models using average performance indices (R2, WI, RMSE, SI, MAE, MAPE, and MBE) scores across 10-folds with the Bayesian optimization (BO) process. The findings from Fig. 14 indicated that, among the non-ensemble methods, the ANN had the highest scores in terms of R2 and WI, followed by the ANFIS and GEP models. Among the ensemble methods, the CatBoost, XGBoost, and RF models demonstrated superior scores in these indices, indicating better overall performance. When evaluating the metrics of RMSE, SI, MAE, and MAPE, it was observed that both ANN and ensemble models demonstrated improved performance by achieving lower values. In addition, although certain models displayed either positive or negative biases, ensemble models, except the AdaBoost model, generally exhibited MBE values that were closer to zero. This suggests a decrease in bias in the predictions developed by the ensemble models.

Upon examining the performance through k-fold cross-validation, it was found that ensemble models outperformed other models in terms of various indices. The CatBoost model consistently demonstrated high R2 and WI values close to 1.0, as well as low values for RMSE, SI, MAE, MAPE, and MBE, which were close to 0.0. Based on the average performance across the tenfold, the CatBoost model stands out as the best-performing model, surpassing the alternatives in terms of accuracy, precision, and reliability.

#### Performance of models across CS ranges

Table 6 provides a comparative analysis of the adopted ML models across three data ranges for the CS: (1) low CS (2.33–25.02), moderate CS (25.08–55.02), and high CS (55.06–82.60). It also focuses on how each model’s accuracy and error indices vary with the complexity of the dataset.

**Table 6:** Comparison of the adopted ML models for different CS ranges.

CS range	Performance indices	MLR	MNLR	SVR	GEP	ANN	ANFIS	RF	AdaBoost	XGBoost	CatBoost
Low CS	R^2^	0.248	0.173	0.271	0.529	0.731	0.411	0.827	0.519	0.842	**0.854**
	RMSE (MPa)	10.05	11.66	9.98	6.10	3.26	6.16	2.73	8.46	2.53	**2.37**
	MAPE	0.614	0.778	0.609	0.364	0.111	0.339	0.111	0.615	0.109	**0.098**
Moderate CS	R^2^	0.211	0.204	0.205	0.450	0.862	0.596	0.925	0.571	0.922	**0.932**
	RMSE (MPa)	9.65	8.97	10.16	6.61	3.19	5.61	2.19	5.48	2.24	**2.08**
	MAPE	0.197	0.178	0.195	0.134	0.042	0.117	0.040	0.113	0.043	**0.041**
High CS	R^2^	0.125	0.001	0.093	0.249	0.744	0.505	0.801	0.414	0.886	**0.925**
	RMSE (MPa)	13.28	15.91	13.32	10.58	4.40	8.11	3.99	8.46	2.95	**2.32**
	MAPE	0.168	0.203	0.167	0.137	0.035	0.103	0.039	0.113	0.029	**0.026**

*The bold values indicated the best predictive models across the ranges of CS.

In the low CS range, the CatBoost, XGBoost, and RF models showed remarkable performance, reaching the highest R^2^ values and the lowest RMSE and MAPE values. These results indicate that these models possess strong predictive accuracy and reliability. The CatBoost model superior to all other ML models, with an R^2^ value of 0.854, an RMSE value of 2.37 MPa, and a MAPE value of 9.80%. The CatBoost, XGBoost, and RF techniques provide the highest accuracy and lowest error metrics, thereby demonstrating their superior predictive capabilities within this range. Although the ANN achieved satisfactory performance with an R^2^ value of 0.731, an RMSE of 3.26 MPa, and a MAPE of 11.10%, its error indices were slightly higher compared to the ensemble models, expect for the AdaBoost model. However, non-ensemble models such as MLR, MNLR, and SVR represent low R^2^ values and high RMSE and MAPE, suggesting limited predictive ability within this range.

For moderate and high CS ranges, the same patterns were observed, with the ensemble models, outperforming the others. Furthermore, the CatBoost model still demonstrated exceptional performance, highlighting its reliable predictive accuracy across different CS ranges. The XGBoost and RF models demonstrate high performance, especially in the moderate and high CS ranges than the low CS range. Although ANN show good performance, particularly in the low and moderate CS ranges, it is surpassed by ensemble models, with the exception of the AdaBoost model. These findings suggest that for applications requiring precise ML predictions in varying CS ranges, the CatBoost and XGBoost models should be considered due to their consistent and superior performance.

#### Rank analysis

A rank analysis is performed to assess the overall performance of the adopted models based on their performance indices calculated in Tables 4 and 5. However, a model overall rank value of 1 represents the best performance and a model value of 10 represents the worst. The overall ranking of each model is determined by summing up the individual ratings. The model with the highest total rank is considered the least effective, whereas the model with the lowest total rank is considered the most effective. Table 7 illustrates the results of the rank analysis in both training (TR) and testing (TS) stages. However, the CatBoost model stands out above all ten models with an overall rank of 20. XGBoost holds the 2nd overall rank with a score of 33. The RF is ranked 3rd overall with a score of 41, followed closely by ANN, ANFIS, and GEP, all sharing the 4th, 5th, and 6th positions with a score of 52, 72, and 82, respectively. However, the AdaBoost, MLR, SVR, and MNLR models are ranked 7th, 8th, 9th, and 10th, respectively, with notably high scores of 101, 114, 121, and 134, respectively.

**Table 7:** Rank analysis of the adopted models.

Model	Stage	R^2^	WI	RMSE	SI	MAE	MAPE	MBE	Total Score	Overall Rank
MLR	TR	8	8	8	9	9	9	6	57	114
	TS	8	8	8	9	8	9	7	57	
MNLE	TR	10	10	10	10	10	10	8	68	134
	TS	10	10	10	10	10	10	6	66	
SVR	TR	9	9	9	8	8	8	9	60	121
	TS	9	9	9	8	9	8	9	61	
GEP	TR	5	7	7	6	7	6	4	42	82
	TS	6	6	6	6	6	5	5	40	
ANN	TR	4	4	4	4	1	1	7	25	52
	TS	4	4	4	3	4	4	4	27	
ANFIS	TR	6	5	5	5	5	5	2	33	72
	TS	5	5	5	5	5	6	8	39	
RF	TR	3	3	3	3	2	2	5	21	41
	TS	3	3	3	4	3	3	1	20	
AdaBoost	TR	7	6	6	7	6	7	10	49	101
	TS	7	7	7	7	7	7	10	52	
XGBoost	TR	2	2	2	2	4	4	3	19	33
	TS	2	2	2	2	2	2	2	14	
CatBoost	TR	1	1	1	1	3	3	1	11	20
	TS	1	1	1	1	1	1	3	9	

### SHAP feature importance

**Figure 15:**
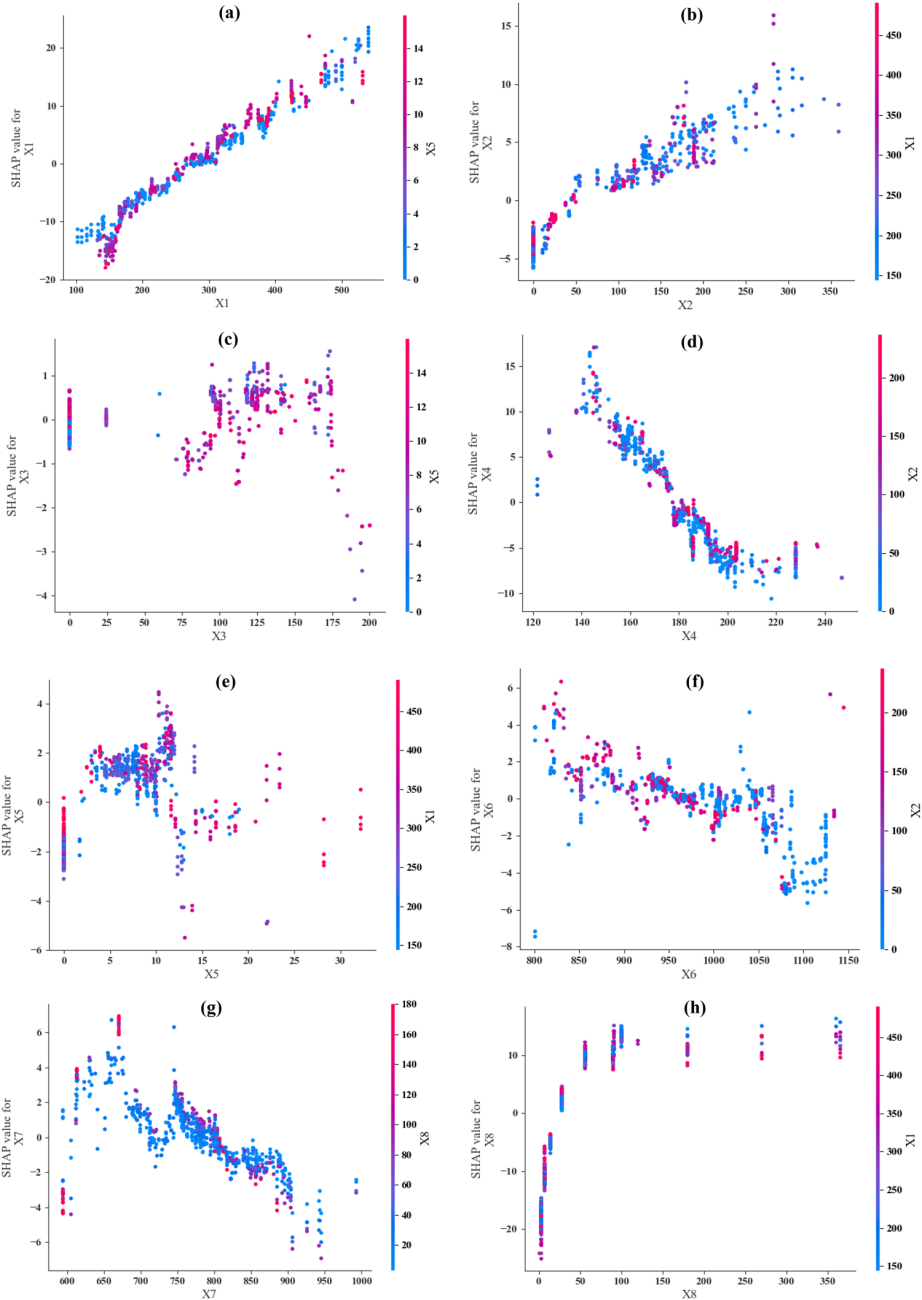
Dependence plots of SHAP value for (**a**) X1 vs. X1 by X5, (**b**) X2 vs. X2 by X1, (**c**) X3 vs. X3 by X5, (**d**) X4 vs. X4 by X2, (**e**) X5 vs. X5 by X1, (**f**) X6 vs. X6 by X2, (**g**) X7 vs. X7 by X8, and (**h**) X8 vs. X8 by X1.

Sensitivity analysis serves to examine data types more closely and to understand the significance of each input parameter in relation to the output^62,90^. The SHAP feature importance results were generated using the best predictive model (CatBoost). Figure 15 shows several dependency charts that highlight the conclusions drawn from the model. The dependence plot’s dots indicate a single dataset prediction. The y-axis designates the appropriate SHAP value, and the x-axis shows the feature value obtained from the features matrix. The SHAP value denotes the degree to which the model’s output for a given prediction is affected by knowing the value of a particular feature. The color mapping in the plot represents a second feature that might interact with the plotted feature.

Figure 15a shows the SHAP value for X1 versus X1, colored by X5. As X1 increases, SHAP values also increase, demonstrating a positive correlation. Higher values of X1 have a more substantial positive impact on the model’s prediction. For the color bar of X5, where data points in red (higher X5 values) correspond to higher SHAP values for X1, suggesting that high X5 values enhance the positive effect of X1 on the prediction. Figure 15b illustrates the SHAP value for X2 versus X2, colored by X1. As X2 increases, SHAP values increase, showing a positive relationship. Higher X2 values have a greater positive impact on the model’s output. Higher X1 values (red) are associated with higher SHAP values for X2, indicating that X1 enhances the positive impact of X2. In Fig. 15c, the SHAP value for X3 versus X3 is shown, colored by X5. The relationship between X3 and its SHAP values is unclear, showing scattered positive and negative impacts. Higher X3 values do not consistently correlate with higher SHAP values. For the color bar of X5, no strong correlation between X5 values and SHAP values for X3. Figure 15d shows the SHAP value for X4 versus X4, colored by X2. As X4 increases, SHAP values decrease, indicating a negative correlation. Higher X4 values have a stronger negative impact on the model’s prediction. For the color bar of X2, higher X2 values (red) correspond to higher SHAP values for X4, suggesting that X2 mitigates the negative effect of X4. Figure 15e presents the SHAP value for X5 versus X5, colored by X1. Increasing X5 leads to mixed SHAP values with both positive and negative impacts. Higher X5 values do not consistently correlate with higher SHAP values. For the color bar X1, higher X1 values (red) show varied impacts on SHAP values for X5. Figure 15f shows the SHAP value for X6 versus X6, colored by X2. As X6 increases, SHAP values generally decrease, indicating a negative correlation. Higher X6 values have a negative impact on the model’s prediction. For the color bar of X2, higher X2 values (red) correlate with higher SHAP values for X6, indicating that X2 mitigates the negative effect of X6. Figure 15g illustrates the SHAP value for X7 versus X7, colored by X8. As X7 increases, SHAP values generally decrease, indicating a negative correlation. Higher X7 values have a negative impact on the model’s prediction. For the color bar of X8, higher X8 values (red) are associated with higher SHAP values for X7, suggesting that X8 mitigates the negative effect of X7. Finally, Fig. 15g shows the SHAP value for X8 versus X8, colored by X1. As X8 increases, SHAP values increase, showing a positive relationship. Higher X8 values have a greater positive impact on the model’s prediction. For the color bar of X1, higher X1 values (red) are associated with higher SHAP values for X8, indicating that X1 increases the positive effect of X8.

In summary, the input parameters of X1, X2, and X8 show positive correlations with their SHAP values, indicating that increases in these features generally enhance the model’s predictions. In contrast, the inputs of X4, X6, and X7 exhibit negative correlations with their SHAP values, indicating that increases in these features generally reduce the model’s predictions. Meanwhile, the inputs of X3 and X5 show mixed or less clear impacts on SHAP values, indicating that their influence on the model’s predictions is inconsistent.

**Figure 16:**
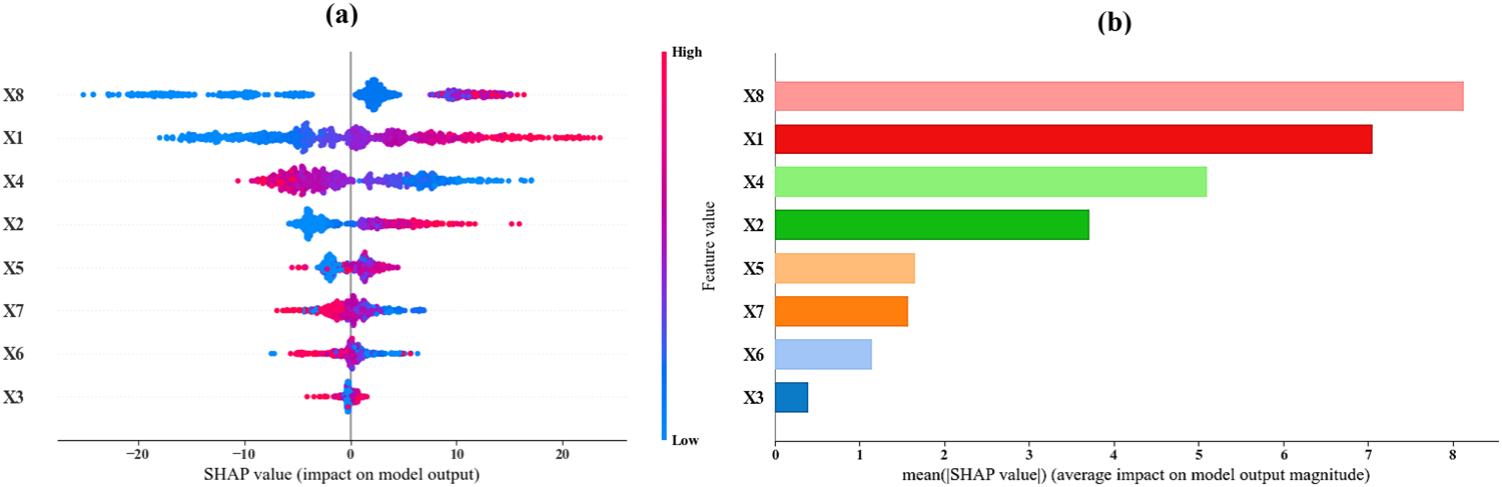
Summary plot of SHAP values for the input variables based on the best predictive model (CatBoost).

Figure 16a presents a summary plot illustrating the distribution of SHAP values for all features based on the best predictive model (CatBoost). On the x-axis, the SHAP value signifies the impact of each feature on the model’s output, while the y-axis lists the inputs. Each dot on the plot represents a single instance, with color coding to indicate feature values—blue for low values and red for high values. For instance, inputs X8 and X1 show a wide range of SHAP values, suggesting it significantly impacts the model’s predictions. Higher values of these inputs tend to correspond with higher SHAP values, indicating a strong positive correlation. Similar patterns are evident for other inputs, where high input values (represented by red dots) generally lead to higher SHAP values, reflecting a positive impact on the model’s output.

Figure 16b depicts the mean absolute SHAP value for each input, highlighting the average impact of each input on the model’s predictions. The higher the mean SHAP value, the more critical the input is to the model. According to this plot, feature X8 (i.e., number of days of curing) has the highest mean SHAP value, marking it as the most important input. This is followed in importance by inputs X1, X4, X2, X5, X7, X6, and finally, X3. Thus, the ranking of inputs based on their importance from highest to lowest is as follows: X8, X1, X4, X2, X5, X7, X6, and X3.

### Comparison with previous studies

The developed ML models were compared to recent similar studies that also aimed to predict the CS of concrete. Table 8 shows the comparative analysis with previous studies for predicting the CS of concrete. Liu^91^, with the XGBoost model, shows an exceptional R^2^ of 0.999, indicating very high accuracy. However, the dataset size is relatively small (60), which may not generalize to larger datasets. Hongwei et al.^19^ found that the bagging regressor was the best predictive model with high R^2^ (0.950) but with higher RMSE and MAE values, likely due to the small dataset size (98). Satish et al.^92^, with R^2^ = 0.950, RMSE = 3.06 MPa, and MAE = 2.13 MPa, performs well on a moderately large dataset (633).

**Table 8:** Comparative analysis for prediction of the CS of concrete.

Reference	Best model	Dataset size	R^2^	RMSE (MPa)	MAE (MPa)
Feng et al. (2020)^93^	AdaBoost	1030	0.94	1.93	1.43
Hongwei et al. (2021)^19^	Bagging regressor	98	0.95	4.97	3.69
Yang et al. (2022)^37^	RF	471	0.89	4.71	3.26
Beskopylny et al. (2022)^94^	KNN	249	0.99	2.62	1.97
Liu (2022)^91^	XGBoost	60	0.999	1.372	–
Li and Song (2022)^95^	GBDT	204	0.942	3.077	2.507
Liang et al. (2023)^86^	BP-GA	190	0.947	5.8	4.49
Satish et al. (2023)^92^	XGBoost	633	0.95	3.06	2.13
Elhishi et al. (2023)^88^	XGBoost	1030	0.91	4.37	3.04
Present study	CatBoost	1030	0.966	3.06	2.27

For larger datasets, the present study developed the CatBoost with R^2^ of 0.966, RMSE = 3.06 MPa, and MAE: 2.27 MPa, demonstrating strong performance on a large dataset (1030). Feng et al.^93^ developed an AdaBoost model that performs well with an R^2^ of 0.940, RMSE of 1.93 MPa, and MAE of 1.43 MPa on a similar dataset size (1030). Also, Elhishi et al.^88^, with R^2^ = 0.910 on a large dataset (1030), show lower accuracy and higher error metrics compared to the present study. The studies with similar dataset sizes, like Feng et al.^93^ and Elhishi et al.^88^, show good performance, but the CatBoost in the present study outperforms them regarding R^2^.

### Interactive graphical user interface (GUI)

**Figure 17:**
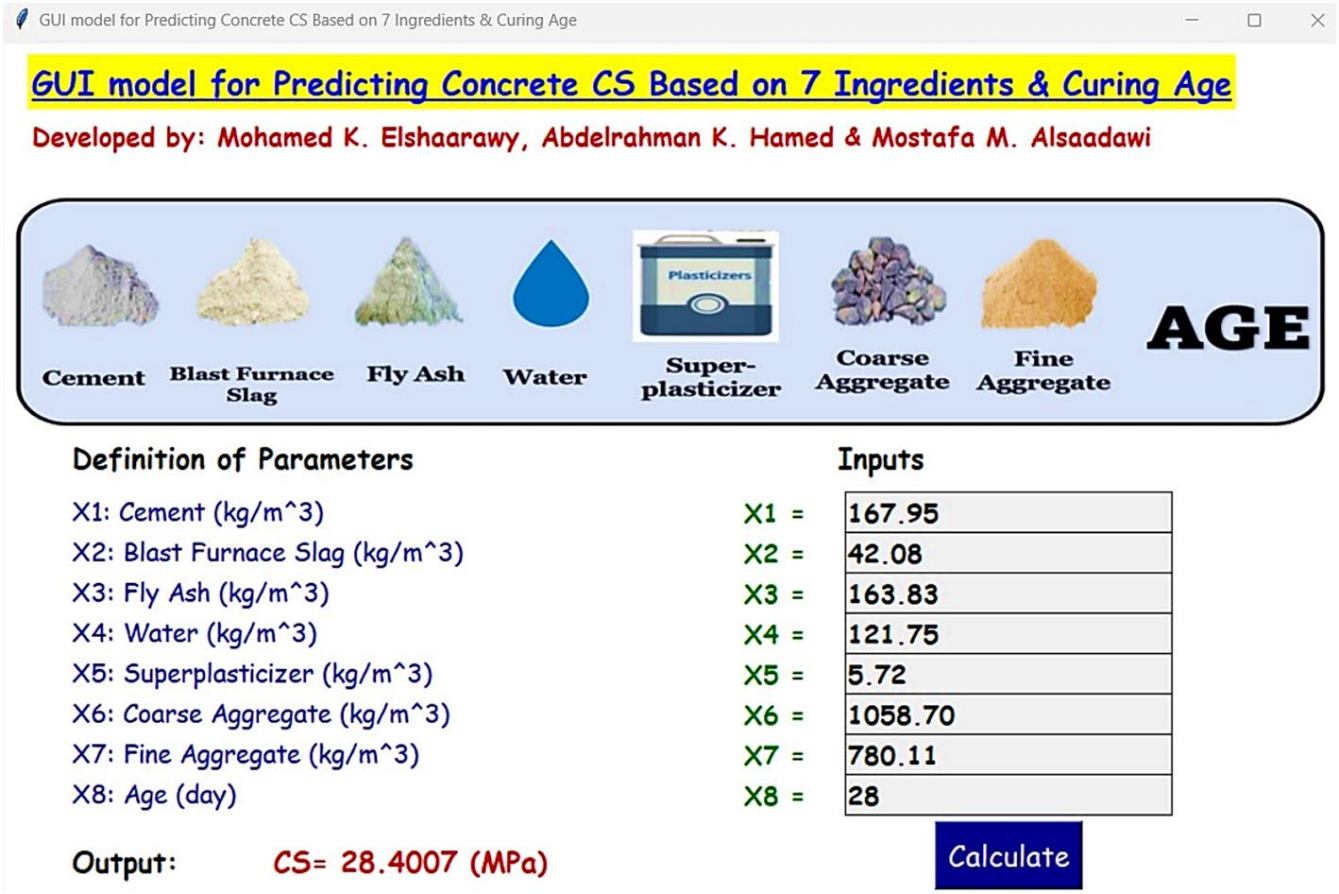
GUI example based on the CatBoost model.

Finally, to address the practical needs of designers in efficiently applying ML models to their needs, this section introduces a significant advancement. Despite the complex requirements of database assembly, model training, and testing hindering the seamless adoption of ML in everyday design tasks, a novel solution has been crafted. A Python web application has been developed, integrating a model with optimized hyperparameters via an intuitive GUI. This GUI is specifically designed to predict concrete CS and streamline the design process, as illustrated in Fig. 17. The CS of concrete value is directly depicted by clicking the “**Calculate**” button. The CatBoost model’s GUI was built to predict the CS value, which was designed with the Tkinkter package^96^. It can be freely accessed at https://github.com/mkamel24/CS.

## Conclusions

In this research, a comprehensive analysis of 1030 data sets on concrete CS was conducted using information gathered from past journal papers. The primary objective of this study was to compare the performance of non-ensemble and ensemble ML models in predicting the CS of concrete containing fly ash. The cement, blast-furnace-slag, fly ash, water, superplasticizer, coarse aggregate, fine aggregate, and curing days were employed as input variables, while the CS was the output variable. The results obtained from both types of models were compared to assess their predictive capabilities. The following key findings emerged from this study:

1. The CatBoost model demonstrated the highest accuracy in predicting CS, with an R^2^ value of 0.966, RMSE of 3.06 MPa, and MAE of 2.27 MPa, outperforming non-ensemble and other ensemble models during the testing stage.
2. A thorough evaluation framework was established incorporating visual methods (scatter plots, violin boxplots, Taylor diagrams) and quantitative methods, along with k-fold cross-validation, to ensure the high reliability and accuracy of the predictions. The CatBoost model was identified as the best-performing model in these evaluations.
3. The analysis revealed that ensemble models, particularly CatBoost, XGBoost, and RF, showed superior performance across low (2.33–25.02 MPa), moderate (25.08–55.02 MPa), and high (55.06–82.60 MPa) CS ranges. Among these, the CatBoost model consistently demonstrated the best performance.
4. Using SHAP analysis, the study identified the most influential parameters affecting CS prediction. The age of the concrete was the most critical factor, followed by cement content. Parameters like water, coarse aggregates, and fly ash had moderate effects, while superplasticizer and fine aggregates were the least significant. The CatBoost model was used for this sensitivity analysis.
5. The CatBoost model in this study demonstrated superior performance on a large dataset compared to similar models in recent studies, confirming its effectiveness and generalizability.
6. To bridge the gap between complex computational predictions and real-world applications, a user-friendly GUI was developed and hosted on an open-source platform like GitHub. Utilizing the CatBoost model, this Python web application allows designers to predict concrete CS quickly and economically. The GUI enhances cost-efficiency and resource management compared to traditional laboratory testing, supports real-time, precise predictive capabilities, and fosters a collaborative environment for ongoing model refinement and improvement.

However, several limitations should be noted. The model’s performance is contingent on the quality and range of the input data. It may not generalize well to extremely different datasets or conditions not represented in the training data. The current model does not account for all possible variables that could affect concrete CS, such as temperature and humidity during curing, which may limit its accuracy in certain scenarios. Additionally, while the GUI is designed for ease of use, it requires users to understand the input parameters and their significance.

Future work should focus on expanding the dataset to include more diverse concrete compositions and external conditions. Incorporating deep learning models could potentially enhance predictive accuracy by capturing more complex relationships within the data. Moreover, the GUI could be improved by integrating more user guidance features and tutorials to assist users with varying levels of expertise. Finally, validating the model with real-world data and scenarios will be essential to ensure its robustness and reliability in practical applications.

## Data Availability

The datasets used and/or analyzed during the current study are available from the corresponding author on reasonable request.
